# Genome-wide profiling of nucleosome sensitivity and chromatin accessibility in *Drosophila melanogaster*

**DOI:** 10.1093/nar/gkv978

**Published:** 2015-10-01

**Authors:** Răzvan V. Chereji, Tsung-Wai Kan, Magda K. Grudniewska, Alexander V. Romashchenko, Eugene Berezikov, Igor F. Zhimulev, Victor Guryev, Alexandre V. Morozov, Yuri M. Moshkin

**Affiliations:** 1 Program in Genomics of Differentiation, *Eunice Kennedy Shriver* National Institute for Child Health and Human Development, National Institutes of Health, Bethesda, MD 20892, USA; 2 Department of Biochemistry, Erasmus Medical Center, P.O. Box 2040, 3000 CA Rotterdam, The Netherlands; 3 European Research Institute for the Biology of Ageing, University of Groningen, University Medical Center Groningen, Groningen, 9713AD, The Netherlands; 4 Institute of Cytology and Genetics, Siberian Branch of RAS, Novosibirsk 630090, Russia; 5 Institute of Molecular and Cellular Biology, Siberian Branch of RAS, Novosibirsk 630090, Russia; 6 Department of Physics and Astronomy and BioMaPS Institute for Quantitative Biology, Rutgers University, Piscataway, NJ 08854, USA

## Abstract

Nucleosomal DNA is thought to be generally inaccessible to DNA-binding factors, such as micrococcal nuclease (MNase). Here, we digest *Drosophila* chromatin with high and low concentrations of MNase to reveal two distinct nucleosome types: MNase-sensitive and MNase-resistant. MNase-resistant nucleosomes assemble on sequences depleted of A/T and enriched in G/C-containing dinucleotides, whereas MNase-sensitive nucleosomes form on A/T-rich sequences found at transcription start and termination sites, enhancers and DNase I hypersensitive sites. Estimates of nucleosome formation energies indicate that MNase-sensitive nucleosomes tend to be less stable than MNase-resistant ones. Strikingly, a decrease in cell growth temperature of about 10°C makes MNase-sensitive nucleosomes less accessible, suggesting that observed variations in MNase sensitivity are related to either thermal fluctuations of chromatin fibers or the activity of enzymatic machinery. In the vicinity of active genes and DNase I hypersensitive sites nucleosomes are organized into periodic arrays, likely due to ‘phasing’ off potential barriers formed by DNA-bound factors or by nucleosomes anchored to their positions through external interactions. The latter idea is substantiated by our biophysical model of nucleosome positioning and energetics, which predicts that nucleosomes immediately downstream of transcription start sites are anchored and recapitulates nucleosome phasing at active genes significantly better than sequence-dependent models.

## INTRODUCTION

Eukaryotic DNA is packaged in the nucleus into a compact nucleoprotein complex called chromatin. The fundamental unit of chromatin is a nucleosome comprising 147 base pairs (bp) of DNA wrapped around a histone octamer core in ≈1.67 left-handed superhelical turns (1). Multiple nucleosomes assemble into one-dimensional ‘beads-on-a-string’ arrays; depending on the organism and cell type, nucleosomes cover 75–90% of genomic DNA (2,3). The ‘beads-on-a-string’ arrays are further folded into higher-order chromatin fibers through intermolecular associations between nucleosomes followed by binding of linker histones H1 and multiple non-histone chromosomal proteins (4–7). Due to tight wrapping of nucleosomal DNA around the core histone octamer and folding into higher-order chromatin structure, accessibility of the genomic DNA to DNA-binding proteins, such as transcription factors (TFs), polymerases and repair enzymes, is largely suppressed (8–12). Thus all cellular genomic functions are mediated by chromatin and typically require modulation of chromatin accessibility.

Nucleosome positioning along the DNA is a crucial factor of chromatin accessibility (13). Thermodynamics and kinetics of nucleosome arrays are thought to result from the balance between intrinsic histone–DNA preferences, statistical positioning and active repositioning of nucleosomes by cellular enzymatic machinery (14–20). Several bioinformatics and biophysical models trained on large-scale nucleosome maps indicate that a ≈10–11 bp periodic distribution of WW (W: A or T) dinucleotides followed in 5–6 bp by SS (S: G or C) dinucleotides signifies an optimal nucleosome formation site (2,21–26). Although performance of such models significantly exceeds random expectations, DNA sequence alone cannot explain all aspects of cellular nucleosome positioning and dynamics. Indeed, insertion of a synthetic nucleosome positioning sequence into the mouse genome resulted in only transient nucleosome formation on this site (27). More generally, substantial differences are observed between *in vivo* and *in vitro* nucleosome occupancy profiles (21,28). Nucleosome dynamics is stimulated *in vivo* by ATP-dependent chromatin remodeling enzymes (remodelers)—abundant nuclear factors which effect nucleosome sliding and unfolding using energy of ATP hydrolysis (10,29,30). Depletion of remodelers impacts the entire nucleosome landscape, with changes in nucleosome placement observed far beyond remodeler binding sites (15,31). Furthermore, precise placement of the +1 nucleosome downstream of transcription start sites (TSS) of actively transcribed genes is likely to be driven by a combined action of basal and sequence-specific transcription factors, RNA polymerase and other chromatin remodeling activities (16). Due to steric exclusion, downstream nucleosomes (+2, +3, etc.) are phased off a potential barrier formed by transcription machinery and +1 nucleosome in a sequence-independent, statistical manner (32–34).

Nucleosome positions are commonly mapped by treating chromatin with microccocal nuclease (MNase), which preferentially digests linker DNA, followed by paired-end sequencing of undigested DNA fragments (MNase-seq) (35). Alignment of the centers of sequenced fragments provides a genome-wide map of nucleosome dyads positions (36). In single-cell organisms, such as budding (*Saccharomyces cerevisiae*) and fission (*S. pombe*) yeast, nucleosome positions can be determined with nucleotide precision using a recently developed chemical cleavage approach (CC-seq) (37–40). Replacement of a wild-type histone H4 gene with the H4S47C mutant allele allows the specific cleavage of DNA at the nucleosome dyad by a hydroxyl radical-catalysed chain scission (38,41). The resulting CC-seq map captures nucleosome positions with high precision. However, the applicability of CC-seq for other organisms is limited due to the fact that in many species the histone H4 gene resides within a large cluster of repeated histone genes. Therefore, in most cases MNase-seq remains a method of choice for nucleosome profiling.

It is often assumed that nucleosomal DNA is protected from cleavage by endonucleases. However, recent reports cast a doubt on this assumption by showing that nucleosomal DNA exhibits varying degrees of sensitivity to MNase digestion (42–46). Here we mapped nucleosome positions in the *Drosophila* genome by chromatin digestion with two different concentrations of MNase. We used a high concentration of MNase (MNase^HIGH^) for complete digestion of chromatin to mononucleosomes, and a low concentration of MNase (MNase^LOW^) for partial digestion. This procedure produced two distinct maps representing MNase-resistant and MNase-sensitive nucleosomes, respectively. Sequences occupied by MNase-resistant and MNase-sensitive nucleosomes differ significantly with respect to their dinucleotide composition. MNase-resistant nucleosomes are assembled on sequences depleted of A/T and enriched in G/C-containing dinucleotides. In contrast, MNase-sensitive nucleosomes are found on A/T-rich sequences. Furthermore, nucleosome occupancy profiles of MNase-resistant, but not MNase-sensitive, nucleosomes can be partially predicted using sequence-dependent models (21,22). MNase-sensitive nucleosomes are found at the genomic loci which were traditionally considered to be nucleosome-depleted. These include enhancers, DNase I hypersensitive sites (DHS) and transcription start (TSS) and termination (TTS) sites. In contrast, MNase-resistant nucleosomes are enriched over coding regions and sites flanking DHS. We observe that MNase-sensitive nucleosomes become resistant upon lowering the temperature of cell cultures by about 10°C, suggesting that increased sensitivity of these nucleosomes to MNase is not due to sequence biases of MNase cleavage. Finally, following previous work (21,22,32,33,39,47–50), we develop a biophysical framework for modeling nucleosome organization around TSS and other nucleosome-phasing loci, and apply it to explain the stereotypical nucleosome phasing pattern observed in the vicinity of active gene promoters.

## MATERIALS AND METHODS

### *Drosophila* and cell culture methods

Flies of Oregon-R strain were reared at 25°C under standard conditions in population cages. Embryos were collected overnight (≈12 h) on grape juice plates supplied with yeast paste. S2 cells were grown in Schneider's media (Invitrogen) supplied with 10% Fetal Calf Serum (Life Technologies) at 27°C, unless specified otherwise.

### Differential MNase-seq/MNase-ChIP-seq and RNA-seq

MNase digestion of formaldehyde cross-linked chromatin of *Drosophila* embryos and S2 cells was performed as described in Supplemental Methods. Libraries for Illumina sequencing of nucleosomal DNA were prepared with Illumina or SureSelect kits and paired-end sequenced (2 × 25 bp or 2 × 50 bp) on Illumina HiSeq 2000. To assess the temperature effects on nucleosome sensitivity/accessibility to MNase, S2 cells were incubated overnight (≈16 h) at 18°C and fixed with formaldehyde prior to MNase digestion. All MNase reactions were performed at 25°C for 10 min.

MNase^HIGH^-ChIP-seq assays were done as described in Supplemental Methods. In brief, following the complete MNase digestion of embryonic chromatin, nucleosomal fragments were precipitated with ChIP-grade antibodies against histone H3 (ab1791, Abcam) or H2B (15), and sequenced using Illumina HiSeq 2000.

For MNase^LOW^-ChIP-seq, embryonic chromatin was partially digested and fractionated on the 5–30% sucrose gradient. Fractions corresponding to mono-nucleosomes were ChIPed with anti-H3 and anti-H2B antibodies and paired-end sequenced on Illumina HiSeq 2000 (Supplemental Methods). RNA-seq for *Drosophila* S2 cells was performed according to the standard Illumina protocols by ServiceXS (the Netherlands).

### Bioinformatics and biophysics analysis of nucleosome positioning

Sequence reads were aligned using Bowtie2 v 2.1 (http://bowtie-bio.sourceforge.net/bowtie2/index.shtml) against BDGP5/dm3 *D. melanogaster* genome.

Data analysis was carried out using combined reads from all available replicates. All average profiles except the nucleosome occupancy profiles were smoothed using a moving average filter with a 21 bp span. All heat maps were smoothed with a 2D Gaussian filter (σ = 3). The MNase^LOW^ 2n profile was constructed using DNA fragments with the length of 320–380 bp, and the two dyads were placed at one-fourth and three-fourths of each DNA fragment. Finally, the sequence-dependent biophysical model was fitted on MNase^LOW^ 1n+2n data. Details of the model construction and fitting can be found in Supplemental Methods.

Nucleosome repeat lengths (NRLs) were estimated by the Fourier analysis of normalized nucleosome dyad profiles. Specifically, the *R* software (https://www.r-project.org) function *fft* was used to compute the discrete Fourier transform of nucleosome dyad arrays, followed by the calculation of absolute magnitudes with the *mod* function. For NRLs of 100–225 bp in length, the resulting magnitudes were linearly interpolated with the step of 0.01 bp using the *approx* function, spline smoothed with the *smooth.spline* function (the smoothing parameter was set to 0.8) and scaled to the maximum magnitude.

## RESULTS

### Genome-wide mapping of MNase-resistant and MNase-sensitive nucleosomes in *Drosophila* embryos

Nucleosomal DNA is not permanently wrapped around histone octamer cores—rather, nucleosomes undergo partial unwrapping at the entry and exit sites due to thermal fluctuations and the action of ATP-dependent chromatin-remodeling enzymes. Moreover, extra DNA may be pulled into the nucleosome and form a loop. Propagation of such DNA loops along the histone octamer surface, accompanied by breaking and re-forming of histone–DNA contacts, may mediate nucleosome translocation (51). Finally, formation of higher-order chromatin structure may impart varying degrees of accessibility to both nucleosome and linker DNA. Thus we expect the accessibility of nucleosomal DNA to digestion by nucleases (e.g. MNase) to vary widely depending on the DNA sequence and other factors that may affect nucleosome and chromatin fiber stability *in vivo*, such as chromatin remodeler activity and temperature.

**Figure 1.**
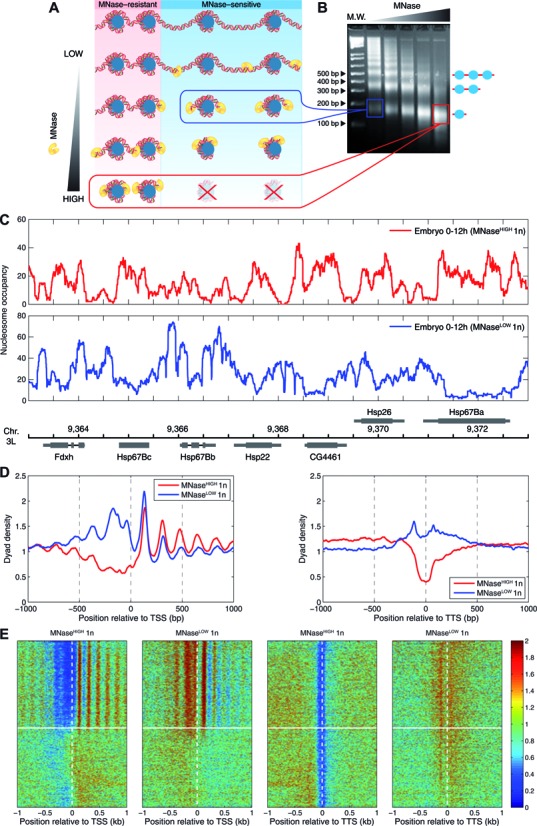
Genome-wide profiling of MNase-sensitive and MNase-resistant nucleosomes in *Drosophila* embryos. (**A**) To map the positions of MNase-sensitive and MNase-resistant nucleosomes, chromatin was digested with varying concentrations of MNase. MNase-sensitive nucleosomes are released upon chromatin digestion with low MNase concentration (MNase^LOW^), and are degraded as MNase concentration is increased. In contrast, MNase-resistant nucleosomes are released only if chromatin is digested with high MNase concentration (MNase^HIGH^). (**B**) Formaldehyde cross-linked chromatin prepared from *Drosophila* 0–12 h embryos was digested with increasing amounts of MNase and loaded onto a 2% agarose gel. DNA fragments corresponding to mono-, di- and tri-nucleosomes are indicated on the right; DNA molecular weight marker (M.W.) is shown on the left. Rectangles indicate mononucleosomal DNA fragments used for further analysis by paired-end sequencing: MNase^LOW^: blue, MNase^HIGH^: red. (**C**) Genomic view of MNase^HIGH^ (upper panel) and MNase^LOW^ (lower panel) nucleosome occupancy profiles derived from paired-end sequencing of mononucleosomal DNA fragments. Genes and genomic coordinates (in kb) are indicated at the bottom. (**D**) Averaged plots of MNase^HIGH^ (red curve) and MNase^LOW^ (blue curve) normalized mononucleosome (1n) dyad profiles aligned at transcription start sites (TSS, left panel) and at transcription termination sites (TTS, right panel). (**E**) Heat maps of MNase^HIGH^ and MNase^LOW^ normalized nucleosome dyad densities around TSS and TTS. Genes were sorted by their expression levels from high (top) to low (bottom) using RNA-seq data for *Drosophila* embryos from modENCODE (http://www.modencode.org/); horizontal white lines separate active and silent genes (71).

To test the idea that nucleosomes exhibit a range of sensitivities to nuclease digestion, we have digested chromatin of 0–12 h *Drosophila* embryos with gradually increasing amounts of MNase (Figure 1A,B). Analysis of DNA fragments obtained with lower MNase concentrations on a 2% agarose gel shows DNA ladders corresponding to mono-, di-, tri-, etc. nucleosome fragments. In contrast, digestion with the highest MNase concentration predominantly yields mononucleosomal DNA fragments (Figure 1B). Moreover, fragment sizes tend to be shorter, consistent with linker DNA trimming and partial digestion of transiently unwrapped ends of nucleosomal DNA by MNase.

Next, from the agarose gel we isolated mononucleosomal DNA obtained with low and high amounts of MNase (blue and red boxes in Figure 1B), and sequenced it using paired-end reads. The average length of DNA fragments is 146.6 bp (replicate 1) and 151.4 bp (replicate 2) with high MNase concentration, with 60.8% and 56.0% of all reads below 147 bp, respectively. The histogram of DNA fragment lengths exhibits secondary peaks corresponding to partially unwrapped nucleosomes (Supplementary Figure S1A). The peaks indicate enhanced DNA accessibility at entry and exit sites of the nucleosome core particle, and are consistent with the idea of stepwise nucleosome unwrapping (39). The secondary peaks are not seen in the histogram of DNA lengths resulting from digestion with low amounts of MNase (Supplementary Figure S1B); the average size of DNA fragments is ≈165 bp, which is likely explained by incomplete linker digestion. The secondary peaks are also absent from the controls in which nucleosome-free genomic DNA was either digested with MNase or sonicated and then paired-end sequenced (Supplementary Figure S1C,D).

We have mapped sequence reads obtained in MNase^HIGH^, MNase^LOW^, and control experiments to the *Drosophila melanogaster* genome, obtaining nucleosome occupancy and dyad density profiles. In nucleosome occupancy profiles, each mate pair represents a single nucleosome, and the number of nucleosomes covering each bp is reported. A representative locus is shown in Figure 1C. In dyad density profiles, dyads (nucleosome centers) are assigned to the midpoint of each mapped nucleosome; raw dyad counts are normalized so that the mean dyad density is 1.0 for each chromosome. Figure 1D shows dyad density profiles in the vicinity of transcription start and termination sites (TSS and TTS, respectively), averaged over all *Drosophila* genes. We observe that MNase-sensitive nucleosomes, whose DNA is overdigested in the MNase^HIGH^ experiment, are strongly enriched immediately upstream of the TSS and across the TTS. In contrast, MNase-resistant nucleosomes are depleted at these loci compared with the chromosome-wide average. This trend is also seen in the heat maps of dyad densities in the vicinity of TSS and TTS (Figure 1E). In addition, genes ordered by their expression levels show marked dichotomy, with highly expressed genes exhibiting much more pronounced nucleosome positioning around the TSS. Interestingly, genomic controls also exhibit oscillations downstream of the TSS, indicating a degree of sequence dependence in MNase digestion and sonication controls (Supplementary Figure S1E). However, the relative magnitude of these oscillations is much smaller and the phase is shifted compared with MNase^HIGH^ chromatin digestion, indicating that the pronounced oscillatory pattern observed in chromatin is not due to experimental biases. Finally, sonication and MNase digestion of genomic DNA exhibit opposite trends around TTS (Supplementary Figure S1F). The magnitude of these trends is again smaller than the difference between MNase^HIGH^ and MNase^LOW^ chromatin digestions.

### Identification of nucleosomal DNA by chromatin immunoprecipitation

It is conceivable that a fraction of the DNA fragments identified by MNase digestion correspond to DNA-bound proteins other than histones, especially in promoters. To alleviate this concern, we have carried out a set of MNase-ChIP-seq experiments (Supplementary Figure S2A). Briefly, chromatin of *Drosophila* embryos was digested as before, using high and low concentrations of MNase. Mono-nucleosomes obtained by the MNase^HIGH^ digestion were precipitated using anti-H2B or anti-H3 antibodies, and the resulting DNA fragments were paired-end sequenced (MNase^HIGH^-ChIP-seq). Mono- and oligonucleosomes obtained by low-MNase digestion were first separated according to their molecular weight by sucrose gradient fractionation. The resulting fractions were analyzed on an agarose gel and a fraction containing mononucleosomes was immunoprecipitated with anti-H2B or anti-H3 antibodies, followed by paired-end sequencing (MNase^LOW^-ChIP-seq). For each antibody and MNase concentration, two independent biological replicates were analyzed. At high MNase concentration, the distribution of fragment lengths is consistent between replicates and does not depend on the antibody used (Supplementary Figure S2B). However, at low MNase concentration both H3 replicates are enriched in short fragments (Supplementary Figure S2C). This is consistent with the presence of partially disassembled nucleosomes, which retained the core H3-H4 tetramer but lost the outer H2A-H2B dimers.

As with the MNase^HIGH^ and MNase^LOW^ data sets, we have mapped MNase^LOW^-ChIP-seq and MNase^HIGH^-ChIP-seq to the *Drosophila* genome, creating nucleosome occupancy and dyad density profiles. Since there is enrichment for short fragments with anti-H3 antibodies, we have divided fragment lengths from H3 experiments into short and long subclasses, with the long subclass window sizes consistent with MNase-seq (Supplementary Figure S2B,C). For ChIP-seq maps, which employed anti-H2B antibodies, we use only the long-fragment subclass. A table of linear correlation coefficients (Supplementary Figure S3A) shows that MNase^HIGH^ and MNase^LOW^ nucleosome occupancy profiles are highly correlated with their ChIP-seq counterparts, regardless of the antibody used. The correlations are strongest when the same fragment lengths are considered and become much weaker when occupancies of short and long fragments are compared. Nevertheless, correlations within MNase^HIGH^ and MNase^LOW^ groups tend to be much higher than the correlations between any two data sets produced using different levels of MNase. We conclude that DNA fragments isolated via MNase^HIGH^-seq and MNase^LOW^-seq come predominantly from nucleosomes. Further, treatments with high and low MNase concentrations isolate distinct subpopulations of MNase-resistant and MNase-sensitive nucleosomes.

After complete digestion, the average dyad density around TSS and TTS is consistent across all data sets (Supplementary Figure S3B). In contrast, after partial digestion there is marked enrichment of short DNA fragments obtained by ChIP-seq with anti-H3 antibodies (orange curve in Supplementary Figure S3C). This enrichment is comparable in magnitude to that observed in the MNase-seq experiment (MNase^LOW^ 1n), and shows that gene promoters are not nucleosome-depleted—rather, they are occupied by MNase-sensitive nucleosomes, a significant fraction of which are partially assembled or partially unwrapped. The enrichment of short-length nucleosomal fragments immediately upstream of the TSS is also visible in the heat maps (Supplementary Figure S3D). Interestingly, the difference between short and long DNA fragments in the MNase^LOW^-ChIP-seq H3 experiment is much less pronounced around TTS, although both are enriched compared to the MNase^LOW^-ChIP-seq H2B nucleosome map.

### MNase-sensitive and MNase-resistant nucleosomes in *Drosophila* S2 cells

Next, we have mapped nucleosomes at high and low MNase concentrations in *Drosophila* Schneider 2 (S2) cells. Unlike a mixture of cells extracted from 0 to 12 h *Drosophila* embryos, S2 cells represent a homogeneous cell type; we expect their gene expression states to be similar across the population. Besides, nucleosome unwrapping and partial disassembly, nucleosome remodeling and chromatin fiber packaging (the primary factors that affect DNA accessibility to MNase) are not limited to embryonic chromatin. Supplementary Figure S4A demonstrates our procedure for extracting nucleosomal DNA from *Drosophila* S2 cells. As with embryos, we have used an agarose gel to analyze DNA fragments obtained at low and high MNase concentrations. In the MNase^HIGH^-seq experiment, we have collected and sequenced mononucleosome-size (1n) fragments, which represent MNase-resistant nucleosomes. The distribution of DNA fragment lengths from paired-end sequencing (Supplementary Figure S4B) is similar to that observed with the embryos (Supplementary Figure S1A). In particular, it exhibits oscillatory substructure indicative of stepwise nucleosome unwrapping and disassembly. In the MNase^LOW^-seq experiment, both mono- and di-nucleosome-sized fragments (1n and 2n, respectively) were isolated and sequenced using paired-end reads (Supplementary Figure S4A). The distribution of 1n fragment lengths (Supplementary Figure S4C) is again similar to that observed in embryonic chromatin (Supplementary Figure S1B), while the lengths of 2n fragments are concentrated in the 300–400 bp range (Supplementary Figure S4D). As with embryos, mononucleosome fragments represent MNase-sensitive nucleosomes, while di-nucleosome fragments should be enriched in MNase-resistant nucleosomes. Indeed, as with embryos the di-nucleosome gel band progressively disappears as MNase concentration is increased, showing that more and more fragments are digested to the mononucleosome level.

We have mapped both 1n and 2n nucleosome fragments to the *Drosophila* genome. Midpoints of 1n fragments were identified with nucleosome dyad positions. For 2n fragments, the dyads were assumed to be located at one-fourth and three-fourth positions with respect to the start of the fragment. MNase^HIGH^ and MNase^LOW^ 1n nucleosome occupancy profiles are reasonably well correlated with their embryonic counterparts, demonstrating that both studies yield similar subsets of MNase-resistant and MNase-sensitive nucleosomes (Supplementary Figure S3A). Furthermore, whereas in S2 cells the MNase^HIGH^ 1n nucleosome occupancy is only weakly correlated with MNase^LOW^ 1n nucleosome occupancy (Pearson's correlation coefficient *r* = 0.39), the correlation with the MNase^LOW^ 2n occupancy profile is much stronger (*r* = 0.69). This observation confirms our view of distinct MNase-resistant and MNase-sensitive nucleosome subsets, and suggests that a combined MNase^LOW^ 1n+2n nucleosome map provides a more comprehensive representation of relative occupancies of all nucleosomes present in *Drosophila* S2 cells.

**Figure 2.**
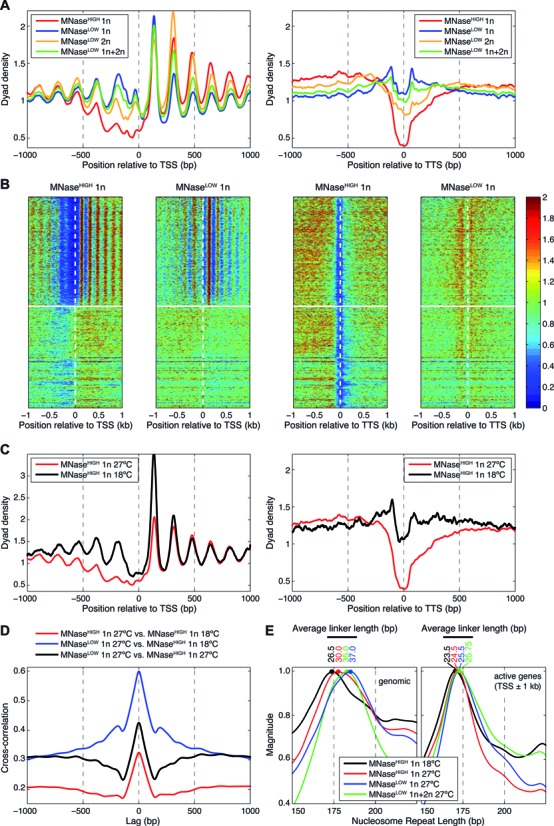
Genome-wide profiling of MNase-sensitive and MNase-resistant nucleosomes in *Drosophila* S2 cells. (**A**) Averaged plots of MNase^HIGH^ mono-nucleosome (1n) and MNase^LOW^ mono-nucleosome (1n), di-nucleosome (2n) and combined (1n+2n) normalized nucleosome dyad profiles aligned at TSS (left panel) and TTS (right panel). (**B**) Heat maps of MNase^HIGH^ 1n and MNase^LOW^ 1n normalized nucleosome dyad densities around TSS and TTS. Genes were ordered by their expression levels from high (top) to low (bottom) using RNA-seq data for *Drosophila* S2 cells (Materials and Methods). Vertical dashed lines mark TSS and TTS positions; horizontal lines separate active and silent genes. (**C**) Averaged plots of MNase^HIGH^ 1n nucleosome dyad profiles aligned at TSS (left panel) and TTS (right panel) for cells grown at two different temperatures: 27°C (red curve) and 18°C (black curve). (**D**) Cross-correlation analysis of MNase^HIGH^ 1n and MNase^LOW^ 1n nucleosome occupancy profiles. The complete digestions (MNase^HIGH^ 1n) were done for cells grown at two different temperatures: 27°C and 18°C. (**E**) Fourier analysis of inter-nucleosome spacing. MNase^HIGH^ 1n at 27°C and 18°C, MNase^LOW^ 1n and MNase^LOW^ 1n+2n normalized nucleosome dyad profiles were Fourier transformed, with Fourier magnitudes spline-smoothed and normalized to the maximum in the 100–225 bp window (see Materials and Methods for details). Average nucleosome linker lengths were determined by subtracting 147 bp from the position of the maximum in the 100–225 bp window. Left panel shows normalized Fourier magnitudes for genome-wide nucleosome dyad profiles; right panel shows Fourier magnitudes in the vicinity of actively transcribed genes (±1000 bp from TSS). Note that the average linker length is shorter in active genes and that, genome-wide, the average linker length is ≈3.5 bp shorter in the MNase^HIGH^ 1n experiment for cells grown at 18°C (black curve) compared with cells grown at 27°C (red curve).

The distributions of dyad densities in the vicinity of TSS and TTS show that, as with chromatin from *Drosophila* embryos, MNase-resistant nucleosomes are depleted in promoters and around TTS (Figure 2A,B). However, this depletion disappears when MNase-resistant and MNase-sensitive nucleosomes are combined into a single profile. This is again due to substantial enrichment of MNase-sensitive nucleosomes in these regions. Since MNase-sensitive nucleosomes disappear as the MNase concentration is increased, chromatin digestion at low MNase concentration followed by sequencing of 1n and 2n nucleosomal fragments provides a more comprehensive representation of *in vivo* nucleosome positioning as compared to MNase^HIGH^ mononucleosome maps alone.

### Temperature affects MNase sensitivity and chromatin accessibility globally

*Drosophila* is a poikilotherm organism that can live normally in temperatures ranging from ≈16°C to 29°C. At the same time, temperature might have a significant impact on chromatin structure, modulating accessibility of genomic DNA. Thus we have studied nucleosome positioning in S2 cells as the cell culturing temperature was changed from 27°C (used in all nucleosome maps thus far) to 18°C (Figure 2C, Supplementary Figure S4B). Following formaldehyde crosslinking, nuclei were isolated and digested with high concentrations of MNase under the same conditions as previously used for MNase^HIGH^ 1n maps. Surprisingly, the low-temperature map of MNase-resistant nucleosomes is better correlated with the high-temperature map of MNase-sensitive nucleosomes (*r* = 0.57 with MNase^LOW^ 1n) than with its high-temperature counterpart (*r* = 0.33 with MNase^HIGH^ 1n) (Supplementary Figure S3A). This observation is consistent with the fact that at 18°C MNase-resistant nucleosomes, many of which are MNase-sensitive at 27°C, are enriched in promoters and around TTS compared to MNase-resistant nucleosomes at 27°C (Figure 2C). These observations are further confirmed by cross-correlation analysis, in which the MNase^HIGH^ 1n profile at low temperature is correlated the most with the MNase^LOW^ 1n profile at high temperature (Figure 2D). This correlation exceeds that between MNase^HIGH^ and MNase^LOW^ 1n profiles at the same temperature (compare blue and black curve in Figure 2D). Thus temperature plays a substantial role in establishing the global patterns of nucleosome sensitivity/accessibility to MNase digestion.

Since the 9°C temperature change is too small to significantly affect intrinsic dynamics and the extent of individual nucleosome unwrapping (k_B_T decreases from 0.60 kcal/mol at 27°C to 0.58 kcal/mol at 18°C), it appears that the marked increase in nucleosome sensitivity to MNase with temperature is mediated primarily through changes in compactness and density of chromatin fibers. Indeed, genome-wide Fourier analysis demonstrates that the average linker length becomes shorter as the temperature is lowered to 18°C, indicating more compact nucleosome placement genome-wide and, to a smaller extent, in the vicinity of active genes (Figure 2E). Since higher-order chromatin structure is very sensitive to linker lengths (52), this change is likely to reflect considerable differences in chromatin fiber folding. Interestingly, around TSS of actively transcribed genes the nucleosome repeat length is ≈5 bp shorter in MNase^HIGH^ conditions and ≈10–11 bp shorter in MNase^LOW^ conditions compared to genome-wide estimates (17). This difference is almost unaffected by lowering the cell culture temperature to 18°C (Figure 2E), suggesting that chromatin structure of active genes is less affected by temperature. Taken together, these differences may explain the varying degree of sensitivity of nucleosomes to MNase.

### Sequence determinants of MNase-sensitive and MNase-resistant nucleosomes

**Figure 3.**
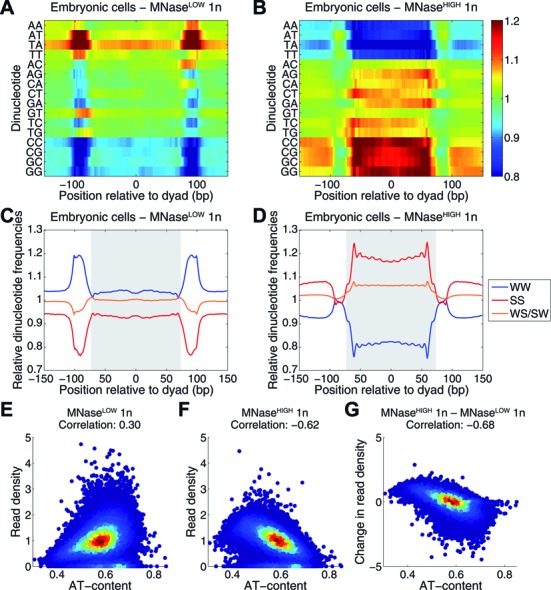
MNase-sensitive and MNase-resistant nucleosomes are associated with distinct sequence motifs. (**A**–**D**) Mononucleosomal DNA fragments from S2 cells were aligned by their centers, and average dinucleotide frequencies were computed and normalized by their genome-wide values. (A) Heat map of relative dinucleotide frequencies with respect to the dyad position, based on the MNase^LOW^ 1n nucleosome map. (B) Same as (A), based on the MNase^HIGH^ 1n nucleosome map. (C,D) Average distributions of WW, SS and WS/SW relative dinucleotide frequencies in nucleosomes from MNase^LOW^ 1n (C) and MNase^HIGH^ 1n (D) experiments. (**E**–**G**) The *Drosophila* genome was tiled into 500-bp non-overlapping windows; the number of reads in each window was divided by its genome-wide average, and for each window the resulting read density was plotted vs. A/T content. (E) MNase^LOW^ mononucleosomes, (F) MNase^HIGH^ mononucleosomes, (G) the difference in read density between MNase^HIGH^ and MNase^LOW^ mononucleosomes. Correlation refers to the linear correlation coefficient between A/T content and (difference in) read density.

We have studied whether MNase-sensitive and MNase-resistant nucleosomes are associated with distinct sequence motifs (such as mono- and di-nucleotide distributions) that might partially explain their genome-wide positioning and varying sensitivity to MNase. We find that in S2 cells mononucleosomal sequences obtained by the MNase^LOW^ assay have frequencies of WW and WS/SW dinucleotides that are close to their genome-wide values, whereas SS dinucleotides are slightly depleted (Figure 3A,C). In contrast, linker DNA is enriched in A/T and depleted in G/C-containing dinucleotides. The situation is almost reversed in the MNase^HIGH^ 1n nucleosome map: mononucleosomal sequences are depleted of A/T and enriched in G/C-containing nucleotides (Figure 3B,D), consistent with previous observations (22,53). Interestingly, the A/T dinucleotides are depleted and G/C dinucleotides are enriched in linkers as well (apart from a narrow region just outside the nucleosome where all dinucleotide frequencies are close to their genome-wide averages). Overall, the observed linker sequence preferences correspond to the fact that MNase preferentially digests A/T-rich sequences (54). Consistent with these findings, read density (defined as the number of reads in 500 bp non-overlapping windows that tile the entire genome) of MNase^LOW^ nucleosomes is only weakly correlated with AT content (Figure 3E), while for MNase^HIGH^ nucleosomes, there is negative correlation (Figure 3F). The difference between MNase^HIGH^ and MNase^LOW^ read densities is positive in genomic regions enriched in MNase-resistant nucleosomes, and negative in genomic regions enriched in MNase-sensitive nucleosomes. This difference is strongly anti-correlated with AT content (Figure 3G), showing that MNase-resistant (and, presumably, more stable) nucleosomes tend to assemble on A/T-poor sequences. The sequence determinants and correlation trends of nucleosome positioning in *Drosophila* embryos are very similar to those observed with S2 cells (Supplementary Figure S5A–G). Thus nucleosome sensitivity to MNase digestion may be partially mediated by DNA sequence.

### Nucleosome stability and chromatin accessibility

**Figure 4.**
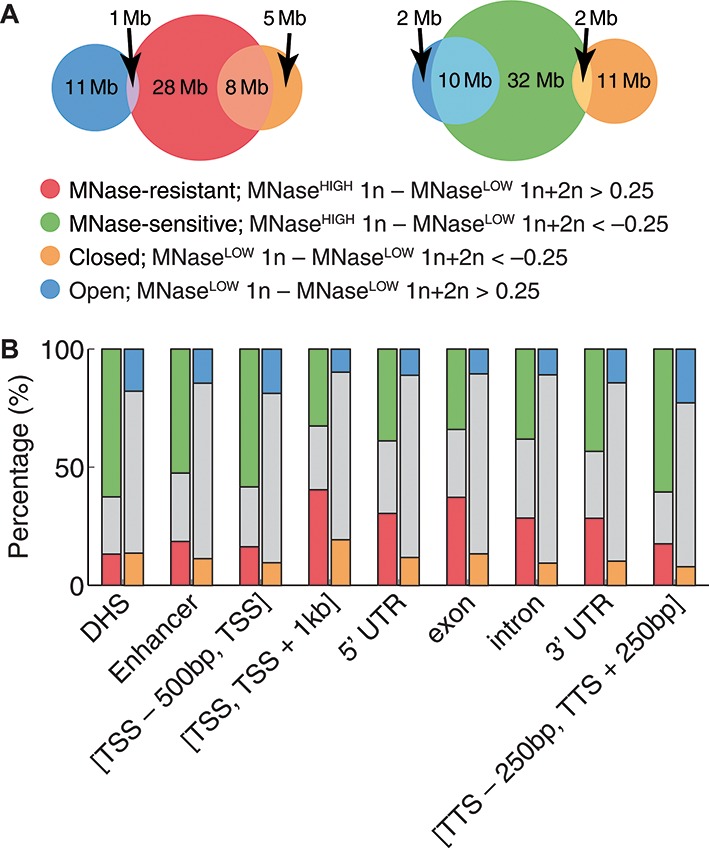
Classification of chromatin from *Drosophila* S2 cells into open/closed and MNase-sensitive/MNase-resistant regions. (**A**) Nucleosome occupancy profiles, normalized to 1.0 for each chromosome, were used to classify genomic bps into MNase-resistant/MNase-sensitive (with MNase^HIGH^ 1n nucleosome occupancy significantly higher/lower than MNase^LOW^ 1n+2n nucleosome occupancy) and open/closed (with MNase^LOW^ 1n nucleosome occupancy significantly higher/lower than MNase^LOW^ 1n+2n nucleosome occupancy) chromatin states. Occupancy threshold of 0.25 was chosen for both classification schemes, as shown in the legend. Note that although the classification is bp by bp, adjacent bps in extended genomic regions tend to have the same label since nucleosome occupancy changes slowly on the bp scale. Venn diagrams show overlaps between MNase-resistant/MNase-sensitive and open/closed regions. We observe that most of the open chromatin is MNase-sensitive, while most of the closed chromatin is MNase-resistant. (**B**) The fraction of bps in MNase-resistant/MNase-sensitive and open/closed categories for several genomic functional regions (gray bars mark the rest of the bps). DHS and enhancer annotations are from (72); all other annotations are from the Flybase (http://flybase.org).

Digestion experiments with high and low MNase concentrations can be used to assess relative accessibility of the chromatin fiber to transcription factors and others DNA-binding proteins. Using MNase^LOW^ 1n, MNase^HIGH^ 1n and MNase^LOW^ 1n+2n nucleosome occupancy profiles (normalized to 1.0 for each chromosome), we classify genomic regions into MNase-resistant/MNase-sensitive and, separately, into open/closed categories (Figure 4). Regions in which MNase^HIGH^ 1n nucleosome occupancy is significantly higher or lower than the total (MNase^LOW^ 1n+2n) occupancy are classified as MNase-resistant or MNase-sensitive, respectively. Likewise, regions in which MNase^LOW^ 1n nucleosome occupancy is higher or lower than the total occupancy correspond to ‘open’ or ‘closed’ chromatin. Indeed, in regions of closed chromatin relatively few mononucleosome-size fragments were obtained at low MNase concentration, and vice versa. The notions of MNase sensitivity and chromatin accessibility are closely related: MNase-resistant chromatin is predominantly closed, while MNase-sensitive chromatin is predominantly open (Figure 4A). Next, we have investigated whether genomic functional elements are enriched in any of the MNase-resistant/MNase-sensitive and open/closed chromatin categories (Figure 4B). We find significant enrichment of DNase I hypersensitive sites (DHS), enhancers, upstream of TSS, and around TTS in MNase-sensitive, open chromatin. In contrast, chromatin downstream of TSS, especially in exons, tends to be classified as MNase-resistant, or closed. Enrichment of MNase-sensitive nucleosomes at DHS suggests that these nucleosomes are equally sensitive to DNase I.

### Chromatin organization and gene expression levels

The distribution of gene expression levels is bimodal in S2 cells (Supplementary Figure S6A). Strikingly, this bimodality is also reflected in the nucleosome organization in the vicinity of coding regions: nucleosomes corresponding to active genes form well-ordered arrays, whereas nucleosomes in and around silent genes are virtually unordered and uniformly distributed (Supplementary Figure S6B,C). The boundary between active and silent genes (horizontal white lines in Supplementary Figure S6C) separates these two distinct types of nucleosome organization. This bimodality is also observed in embryonic gene expression levels and, correspondingly, in embryonic chromatin organization (horizontal white lines in the TSS panels of Figure 1E). Thus nucleosome ordering is strongly affected by the presence of transcriptional machinery in the vicinity of active gene promoters.

### Nucleosome positioning and histone turnover rates

Next, we have investigated whether nucleosome organization is correlated with histone turnover rates (55,56). We have focused on active genes from Supplementary Figure S6A, ordering them by histone turnover rates averaged over the (TSS, TSS+250) region (Supplementary Figure S7A). The ranking of average turnover rates is mostly determined by the difference in the turnover rates of the +1 nucleosome (Supplementary Figure S7C). We have divided all active genes into three equal-size tertiles with high, medium and low turnover rates. The distributions of nucleosome occupancy are qualitatively similar in all three tertiles (Supplementary Figure S7B); however, in the first tertile with the highest turnover rates (Supplementary Figure S7C,F) the +1 nucleosome peak is more pronounced when MNase-sensitive nucleosomes are considered (Supplementary Figure S7B, middle panel). Interestingly, genome-wide maps of Pol II binding obtained using either GRO-seq (Supplementary Figure S7D,G) or ChIP-seq (Supplementary Figure S7E,H) (57) show that polymerase occupancy at the start of coding regions increases with histone turnover rates. Thus the tertile with the highest Pol II occupancy also has the most pronounced, MNase-sensitive +1 nucleosomes. Since Pol II is expected to disrupt nucleosome organization, it is reasonable to assume that chromatin remodelers arrange nucleosomes into ordered arrays in a Pol II dependent fashion, perhaps through remodeler recruitment by polymerases. At the same time, active remodeling affects turnover rates of +1 nucleosomes. Overall, our observations show that the +1 nucleosome is both mobile and more accessible to MNase, strongly suggesting that it is actively remodeled.

### Nucleosome organization in the vicinity of DNase I hypersensitive sites

Nucleosomes are organized into periodic arrays in the vicinity of active gene promoters (Supplementary Figure S6C), likely through interactions with chromatin remodelers, components of transcriptional machinery and regulatory factors. One simple mechanism of nucleosome organization is ‘phasing’ off a barrier created by a DNA-bound factor with sufficiently high binding affinity, so that it is not displaced through thermodynamic competition with nucleosomes (58). Another potential mechanism is through stabilization of one of the nucleosomes, which then serves to anchor the entire nucleosome array. For example, the +1 nucleosome might be stabilized through favorable contacts with RNA polymerase subunits (28).

**Figure 5.**
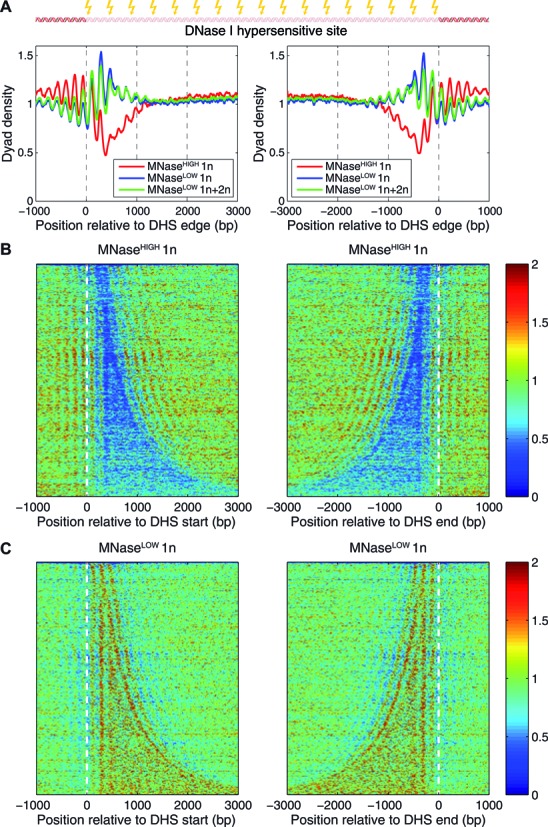
Nucleosome organization in the vicinity of DNase I hypersensitive sites (DHS). (**A**) Averaged plots of MNase^HIGH^ 1n (red curve), MNase^LOW^ 1n (blue curve) and combined MNase^LOW^ 1n+2n (green curve) normalized nucleosome dyad density profiles aligned at left and right edges of DNase I hypersensitive sites (DHS) (72). (**B**,**C**) Heatmaps of MNase^HIGH^ 1n (B) and MNase^LOW^ 1n (C) normalized nucleosome dyad densities around left and right DHS edges. DHS were ordered by length (increasing top to bottom). There are 7374 annotated DHS (72), out of which 3871 (52.5%) contain a TSS and 3051 (41.4%) are at least 1 kb away from any TSS, including 1769 (24.0%) that are at least 5 kb away from any TSS. If only active genes are considered, 3579 (48.5%) DHSs overlap with their TSS, 3504 (47.5%) are at least 1 kb away and 2380 (32.3%) are at least 5 kb away.

If nucleosomes phase off DNA-bound particles, nucleosome arrays should be observed not only in the vicinity of TSS but also genome-wide, wherever DNA-bound factors are located. To investigate this hypothesis, we have studied nucleosome organization in the vicinity of DNase I hypersensitive sites (DHS), which correspond to regions of open chromatin enriched in DNA-bound factors (Figure 5; note that approximately half of all DHS are at least 1 kb away from the nearest TSS). Indeed, we observe striking periodicity in nucleosome density profiles upstream, downstream and inside of DHS (Figure 5A). Moreover, MNase-resistant (MNase^HIGH^ 1n) nucleosomes are depleted inside DHS (Figure 5A,B), likely because competition with non-histone DNA-binding factors makes nucleosomes less stable and thus more sensitive to MNase. Indeed, DHS are enriched with MNase-sensitive (MNase^LOW^ 1n) nucleosomes (Figure 5C). The phasing is likely mediated by interactions (such as steric exclusion) between factors bound in DNase I hypersensitive regions and neighboring nucleosomes.

### Biophysical model of nucleosome positioning

**Figure 6.**
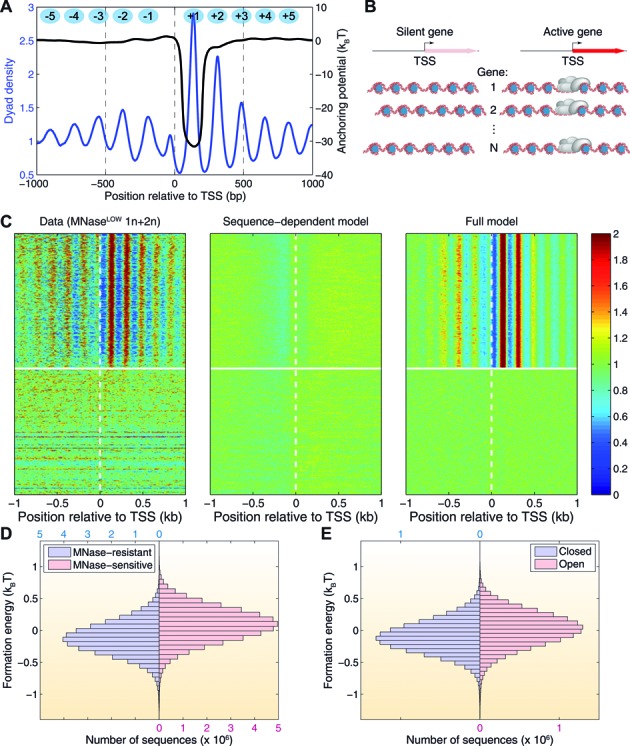
Biophysical nucleosome positioning model. (**A**) Nucleosomes are phased near TSS of active genes. The average normalized nucleosome dyad density in the vicinity of active gene promoters is shown as a blue line, and the effective nucleosome anchoring potential that generates this nucleosome distribution is shown as a black line. The anchoring potential (nucleosome formation energy) at *x* corresponds to a nucleosome with the dyad at position *x*. The potential well at the +1 nucleosome position indicates that the +1 nucleosome in active genes is anchored to the DNA by external factors in a transcription-dependent way. (**B**) Cartoon representing disordered arrays of nucleosomes on silent genes and phased arrays of nucleosomes on active genes. The position of the first nucleosome in the array is stabilized through interactions with other DNA-binding proteins, such as components of transcriptional machinery or chromatin remodeling factors. (**C**) Heat map representing the combined nucleosome dyad density in *Drosophila* S2 cells (MNase^LOW^ 1n+2n; left panel) and the density predicted using a sequence-dependent model (Supplemental Methods; middle panel) and a full model which takes into account both the sequence-dependent contribution to the nucleosome formation energies and the sequence-independent contribution of the external anchoring potential shown in (A) (Supplemental Methods; right panel). The genes are sorted according to their expression levels as in Supplementary Figure S6A; horizontal white lines separate active and silent genes. (**D**,**E**) Histograms of nucleosome formation energies predicted using the sequence-dependent model (Supplemental Methods), in four chromatin regions defined in Figure 4: MNase-resistant/MNase-sensitive (D) and closed/open (E). Nucleosomes in MNase-resistant/closed regions have lower formation energies than nucleosomes in MNase-sensitive/open regions. The energies are reported relative to the genome-wide average nucleosome formation energy. The absolute magnitude of the difference in average energies is 0.21 k_B_T for MNase-resistant and MNase-sensitive nucleosomes (D) and 0.12 k_B_T for nucleosomes in closed and open chromatin regions (E).

Based on our observation that nucleosomes are phased in the vicinity of active genes and DHS, we have developed a model of nucleosome positioning in which interactions with DNA-bound factors and components of transcriptional machinery play a dominant role in active genes, with additional refinements due to sequence-dependent effects (Figure 6). In contrast, in silent genes nucleosomes are positioned primarily by sequence, where little phasing is observed (Figures 1E, 2B and 6C). We have first determined how much of the nucleosome positioning signal can be captured by modeling sequence effects alone. Following our previous work (22,32,33,39), we have fitted a simple model to the MNase^LOW^ 1n+2n data set in which nucleosome formation energy is a function of the mono- and dinucleotide content at each nucleosomal site. Our biophysical model relies on a closed-form solution, which allows us to infer nucleosome formation energies directly from the observed dyad density profile (Supplemental Methods). Briefly, the model exploits the analogy between nucleosome arrays and a one-dimensional fluid of particles with steric exclusion, subject to an arbitrary external field (59). The inferred histone–DNA interaction energy profile is then interpreted in terms of sequence features by means of a linear fit (22). This two-step approach has previously allowed us to explore a hierarchy of models of nucleosome energetics of increasing complexity. We have found that a simple model, which depends only on the mono- and dinucleotide content of the nucleosomal sequence, performs as well as more complex ones with many additional parameters (22). This is the model we employ in this work.

By itself, the sequence-dependent model is unable to predict periodic nucleosome arrays observed in active genes (middle panel in Figure 6C). In other words, the observed periodic variation in nucleosome dyad density is not strongly correlated with sequence features. As a result, the standard deviation of sequence-dependent energies is less than 1 k_B_T in the vicinity of TSS (60). These relatively small energy changes are unable to phase nucleosomes, as can be seen in silent genes, where the nucleosomes are likely positioned by sequence alone and as a result no phasing is observed (left panel in Figure 6C; silent genes are below the horizontal white line). Thus, in order to explain active gene phasing, we introduce a sequence-independent contribution to the total energy, which models interactions between histones and components of transcriptional machinery. First, we construct a nucleosome dyad density profile averaged over all active genes (blue line in Figure 6A). From this average distribution of nucleosome dyads, we infer the corresponding sequence-independent energy profile through an exact calculation (Supplemental Methods; black line in Figure 6A). This calculation reveals that the observed phasing in active genes is due to a potential well centered on the +1 nucleosome. The well makes the +1 nucleosome precisely positioned: the depth of the well is much greater than 1 k_B_T, making it the dominant contribution to the +1 nucleosome energetics. The +1 nucleosome in turn anchors an entire array of upstream and downstream nucleosomes.

The overall mechanism of nucleosome positioning in the vicinity of coding regions is illustrated in Figure 6B. In silent genes, nucleosome positioning may be affected by both sequence and chromatin remodelers. However, in the absence of nucleosome anchoring and since sequence specificity of nucleosome positioning is modest, nucleosome arrays are dynamic and phased differently from gene to gene and from cell to cell, resulting in the lack of overall phasing. In active genes, the +1 nucleosome is strongly positioned with respect to TSS, serving as a nucleation site for phased nucleosome arrays. Indeed, a full model in which the sequence-independent energy profile from Figure 6A is added to sequence-dependent energies succeeds in reproducing the salient features of the nucleosome dyad density profile in both active and silent genes (Figure 6C, right panel). To gauge the predictive power of our models genome-wide, we have computed linear correlation coefficients between experimentally observed dyad densities and nucleosome occupancies and predictions of the sequence-dependent, sequence-independent, and full models in the vicinity of active gene TSS (Supplementary Figure S8). Since exact histone concentrations (or, equivalently, chemical potentials of histone octamers) are not known and may vary during cell cycle and from cell to cell, we change the chemical potential over a reasonable range. Within this range, the sequence-independent model outperforms the sequence-dependent one, and is generally outperformed by the full model. As expected, nucleosome occupancies are predicted with higher accuracy than exact dyad positions.

We expect MNase-resistant nucleosomes to be more energetically stable than MNase-sensitive ones. Indeed, less stable nucleosomes should be more mobile during chromatin remodeling and more susceptible to partial DNA unwrapping, making it easier for nucleases to gain access to nucleosomal and linker DNA. Using our predicted sequence-dependent nucleosome formation energies (Supplemental Methods), we find that MNase-resistant nucleosomes are indeed more stable (Figure 6D). Note that this prediction is based on the combined MNase^LOW^ 1n+2n data set, which contains both MNase-resistant and MNase-sensitive nucleosomes and is therefore less biased toward a particular nucleosome subset compared with MNase^LOW^ 1n and MNase^HIGH^ 1n data sets. Furthermore, because regions with MNase-resistant nucleosomes tend to be classified as closed chromatin, nucleosomes in closed chromatin regions are more stable on average than nucleosomes corresponding to open chromatin (Figure 6E).

## DISCUSSION

Nucleosomes are dynamic structures—they can be unfolded, relocated or partially unwrapped, with the help of chromatin remodeling enzymes or by thermal fluctuations alone (51,61–64). In addition, nucleosome arrays fold into chromatin fibers, which in turn form intricate, dynamic higher-order structures (65). Thus the degree of accessibility of nucleosomal DNA to nucleases such as MNase can vary significantly depending on how mobile the nucleosome is and whether it resides within open or closed chromatin. Multiple factors may contribute to nucleosome mobility, including DNA sequence, chromatin remodeler action and interactions with non-histone DNA-binding proteins such as components of transcriptional machinery (28,31,47,66–68). Such interactions may both prevent nucleosome formation by steric exclusion and facilitate it by establishing favorable contacts with the histone octamer and/or nucleosomal DNA.

Here we show that nucleosomes exhibit a wide range of sensitivities to MNase (43,45,46), and thus MNase concentration determines which nucleosomes will be preferentially isolated in nuclease digestion experiments. Indeed, at higher MNase concentrations typically used in nucleosome mapping experiments to reduce chromatin to mononucleosomes, only MNase-resistant nucleosomes will yield DNA of approximately mononucleosomal length, whereas DNA of MNase-sensitive nucleosomes will be overdigested and lost from the mononucleosomal band. In contrast, at lower MNase concentrations the mononucleosome-size DNA fragments will be contributed primarily by MNase-sensitive nucleosomes, whereas MNase-resistant nucleosomes will correspond to longer (di-, tri-nucleosome, etc.) DNA fragments. Consistent with this idea, we have found that digestions at high and low MNase concentrations yield distinct nucleosome subsets in both 0–12 h *Drosophila* embryos and S2 cells (Supplementary Figure S3A), with MNase-sensitive and MNase-resistant nucleosomes exhibiting unique sequence signatures (Figure 3, Supplementary Figure S5). Moreover, many of the mononucleosome-size fragments are shorter than the canonical nucleosomal DNA length of 147 bp, indicating that, similarly to baker's yeast (39), a significant fraction of fly nucleosomes is partially unwrapped and overdigested by MNase. We have used chromatin immunoprecipitation against H3 and H2B histones to confirm that these shorter DNA fragments, as well as all other fragments analyzed by MNase^HIGH^- and MNase^LOW^-seq, are associated with histone proteins.

Finally, we observe that the broad nucleosome-depleted regions upstream of TSS (69) are actually enriched in MNase-sensitive nucleosomes; these nucleosomes are not detected at higher MNase concentrations. In order to provide a more comprehensive map of nucleosome positioning, we have used both mono- and di-nucleosome fragments isolated at low MNase concentration in S2 cells. Mononucleosome fragments correspond to MNase-accessible nucleosomes that are easily released from chromatin, while di-nucleosome and longer fragments contain nucleosomes from regions that are more inaccessible to MNase. We note that DNA fragments corresponding to more than two nucleosomes (and thus related to inaccessible chromatin regions) were not sequenced (Supplementary Figure S4A). Nonetheless, our analysis goes beyond a standard approach of identifying mononucleosomes at high MNase concentration, providing a more comprehensive picture of *Drosophila* chromatin.

Remarkably, we find that nucleosomes that were MNase-sensitive when cells were grown and harvested at 27°C become MNase-resistant when the temperature is lowered to 18°C (Figure 2). Since this temperature change is too small to significantly affect the scale of thermal fluctuations, we hypothesize that the observed loss of DNA accessibility to MNase is due to changes in higher-order chromatin structure rather than thermally-activated dynamics of individual nucleosomes. This is consistent with our observation that regions of open and closed chromatin are enriched in MNase-sensitive and MNase-resistant nucleosomes, respectively (Figure 4). It is also possible that lowering the temperature modifies enzymatic activity of chromatin remodelers, impeding nucleosome translocation, unfolding and histone exchange and making nucleosomes less susceptible to nuclease digestion.

Sorting genes by expression levels reveals striking bimodality in nucleosome organization around TSS: in active genes we observe phased arrays of well-positioned nucleosomes, whereas in silent genes phasing with respect to TSS is lost (Figure 1, Supplementary Figure S6). This observation is consistent with an idea that nucleosome arrays in the vicinity of active genes are anchored through interactions with regulatory factors and components of transcriptional machinery (28). In contrast, nucleosomes in silent genes are positioned primarily through intrinsic sequence specificity of histone–DNA interactions. The range of sequence-specific nucleosome formation energies observed with genomic DNA is likely to be too small to provide strong phasing of nucleosome arrays, resulting in nucleosome fluid rather than a crystal-like array of well-positioned nucleosomes (60). Interestingly, nucleosomes are also strongly phased in the vicinity of DHS (some of which are far from any coding regions) (Figure 5). This phasing likely results from interactions between nucleosomes and factors bound at the hypersensitive sites, which may create potential barriers and wells on the nucleosome free energy landscape through favorable contacts with nucleosomes and steric exclusion (24,58).

These observations were used to construct a biophysical model of nucleosome distribution in genic regions, in which the +1 nucleosome immediately downstream of the TSS is positioned through interactions with chromatin remodelers mediated by components of transcriptional machinery in active genes (Figure 6). The other nucleosomes are then positioned by steric exclusion, with sequence-dependent effects and active remodeling providing additional refinements (24,47,48,50,70). In silent genes, sequence-dependent positioning and active remodeling are unable to phase nucleosomes in the absence of +1 nucleosome anchoring, which serves to nucleate the array. Overall, our model is successful in reproducing patterns of nucleosome occupancy in both active and silent genes.

Interestingly, the +1 nucleosome, which in our framework anchors the entire array, also exhibits the highest histone turnover rates (Supplementary Figure S7). Histone exchange rates and sensitivity to MNAse at the +1 nucleosome correlate with RNA Pol II occupancy in the vicinity of TSS. Thus it appears that the presence of RNA polymerase both stabilizes the +1 nucleosome, precisely defining its position to a degree not achievable with DNA sequence alone, and at the same time increases its histone exchange rates. The latter requires an active remodeling mechanism since thermally-activated histone exchange should be impeded if the nucleosome is stabilized through interactions with external factors such as Pol II.

## ACCESSION NUMBERS

MNase-seq sequencing data have been deposited to NCBI GEO under accession number GSE69336.

## Supplementary Material

SUPPLEMENTARY DATA
