# Supplementary material for: Genome-wide profiling of nucleosome sensitivity and chromatin accessibility in Drosophila melanogaster
Source: Nucleic Acids Res. 2015 Oct 1;44(3):1036–51. doi: 10.1093/nar/gkv978 (PMC4756854; doi:10.1093/nar/gkv978)
Supplement: SUPPLEMENTARY DATA [file supp_44_3_1036__index.html]

Genome-wide profiling of nucleosome sensitivity and chromatin accessibility in Drosophila melanogaster — Genome-wide profiling of nucleosome sensitivity and chromatin accessibility in Drosophila melanogaster — SUPPLEMENTARY DATA 

# Genome-wide profiling of nucleosome sensitivity and chromatin accessibility in *Drosophila melanogaster*

## SUPPLEMENTARY DATA

- SUPPLEMENTARY DATA
