# Supplementary material for: Genome-wide profiling of nucleosome sensitivity and chromatin accessibility in Drosophila melanogaster
Source: Nucleic Acids Res. 2015 Oct 1;44(3):1036–51. doi: 10.1093/nar/gkv978 (PMC4756854; doi:10.1093/nar/gkv978)
Supplement: SUPPLEMENTARY DATA [file supp_gkv978_nar-01705-m-2015-File008.pdf]

Supplemental Material

Genome-wide profiling of nucleosome sensitivity and chromatin accessibility in *Drosophila melanogaster*

Răzvan V. Chereji[1,*], Tsung-Wai Kan[2,*], Magda K. Grudniewska[3], Alexander V. Romashchenko[4], Eugene Berezikov[3], Igor F. Zhimulev[5], Victor Guryev[3], Alexandre V. Morozov[6,†], and Yuri M. Moshkin[2,4,5,†]

[1]Program in Genomics of Differentiation, *Eunice Kennedy Shriver* National Institute for Child Health and Human Development, National Institutes of Health, Bethesda, Maryland 20892, USA
[2]Department of Biochemistry and Centre for Biomedical Genetics, Erasmus University Medical Center, Rotterdam, The Netherlands
[3]European Research Institute for the Biology of Ageing, University of Groningen, University Medical Center Groningen, Groningen, 9713AD, The Netherlands
[4]Institute of Cytology and Genetics, Siberian Branch of RAS, Novosibirsk 630090, Russia
[5]Institute of Molecular and Cellular Biology, Siberian Branch of RAS, Novosibirsk 630090, Russia
[6]Department of Physics and Astronomy and BioMaPS Institute for Quantitative Biology, Rutgers University, Piscataway, NJ 08854, USA
[*]These authors contributed equally to this work.
[†]Corresponding authors. E-mails: morozov@physics.rutgers.edu (A.V.M.), yury.moshkin@gmail.com (Y.M.M.)

Contents

Supplemental Methods **3**
Statistical Mechanics of Nucleosome Positioning 3
Forward Problem 3
Inverse Problem 5
Energy models 7
Sequence-independent minimal model 7
Sequence-dependent model 7
Full model 9
Differential MNase-seq 10
Differential MNase-ChIP-seq 10

Supplemental Figures **12**
Figure S1 12
Figure S2 13
Figure S3 14
Figure S4 16
Figure S5 17
Figure S6 18
Figure S7 19
Figure S8 21

Supplemental Tables **22**
Table S1 22

Supplemental References **23**

Supplemental Methods

Statistical Mechanics of Nucleosome Positioning and Energetics

Following previous work by us (Locke et al., 2010; Chereji and Morozov, 2011; Chereji et al., 2011; Chereji and Morozov, 2014) and other groups (Chevereau et al., 2009; Teif and Rippe, 2009; Vaillant et al., 2010; Teif and Rippe, 2010, 2012; Mobius et al., 2013; Rube and Song, 2014), we have developed a biophysical approach to predicting nucleosome positioning and energetics from high-throughput sequencing datasets. We assume that a nucleosome consists of 147 base pairs (bp) of DNA wrapped around a histone octamer composed of two copies of four core histones: H2A, H2B, H3 and H4. We model nucleosome arrays as interacting hard rods of length $a = 147$ bp that can be reversibly adsorbed to a one-dimensional lattice with L bps, *e.g.* representing a chromosome.

There are two sets of related quantities to consider: the nucleosome distribution (probabilities of finding the system in every possible configuration), and nucleosome formation energies that dictate this distribution.

Forward Problem

If all interactions within the system are given but the nucleosome distribution is unknown, we have to solve a forward problem. We denote by $u(i)$ the formation energy of a nucleosome with its center (dyad) at position i. This energy may have contributions from DNA bending, electrostatic interactions, hydrogen bond formation, van der Waals forces, etc. Particles are prevented from forming outside of the lattice ends, so that all nucleosomes must be inside the $[0, L]$ range:

$$u(i) = \infty \qquad \text{for all } i < \frac{a}{2}, \text{ and } i > L - \frac{a}{2}.$$

Nucleosomes cannot overlap, and we assume that the neighboring particles interact through a hard-core interaction:

$$\Phi(j,k) = \begin{cases} \infty & \text{if } k - j < a, \\ 0 & \text{otherwise.} \end{cases}$$

Here, j and k indicate the positions of neighboring nucleosome dyads, and $\Phi(j,k)$ represents the two-body interaction between the two particles. Two-body interactions may also be used to account for the effects of higher-order chromatin structure (Chereji et al., 2011; Chereji and Morozov, 2011).

For a fixed number of particles bound to DNA, N, the canonical partition function (Reichl, 2009) is

$$Q_N = \sum_{\{i_n=1\ldots L\}_{n\in\{1,\ldots,N\}}} e^{-\beta u(i_1)} e^{-\beta\Phi(i_1,i_2)} e^{-\beta u(i_2)} \ldots e^{-\beta u(i_{N-1})} e^{-\beta\Phi(i_{N-1},i_N)} e^{-\beta u(i_N)}, \tag{1}$$

where β is the inverse temperature: $\beta = 1/(k_B T)$ (k_B is the Boltzmann constant, and T is the temperature). Our definition of the two-body interaction guarantees that only valid configurations of non-overlapping nucleosomes contribute to Eq. (1).

If the system is allowed to have a variable number of particles (*e.g.* different cells may have a different total number of nucleosomes on the same chromosome), the grand-canonical ensemble formalism (Reichl, 2009) is more appropriate, and the partition function is

$$Z = \sum_{N=0}^{N_{\max}} e^{\beta N\mu} Q_N,$$

where μ is the chemical potential, and $N_{\max}$ is the maximum number of non-overlapping rods that can fit in L bp. Note that any configuration with a number of particles $N > N_{\max}$ does not contribute to Z, because in this case at least two particles overlap. This allows us to extend the upper limit from $N_{\max}$ to ∞. From the partition function we can compute s-particle distribution functions,

$$n_s(i_1, \ldots, i_s) = \frac{\zeta(i_1) \ldots \zeta(i_s)}{Z} \frac{\delta^s Z}{\delta\zeta(i_1) \ldots \delta\zeta(i_s)},$$

where

$$\zeta(i) = e^{\beta[\mu - u(i)]}.$$

In particular, the one-particle distribution is given by

$$n_1(k) = \frac{\zeta(k)}{Z} \frac{\delta Z}{\delta\zeta(k)},$$

which represents the probability of finding a nucleosome with its dyad located at position k. From this we obtain the probability of finding bp i covered by any nucleosome, or nucleosome occupancy:

$$\text{Occ}(i) = \sum_{k=i-\lfloor a/2 \rfloor}^{i+\lfloor a/2 \rfloor} n_1(k),$$

where

$$\lfloor a/2 \rfloor = \max\{i \in \mathbb{N} | i \leq a/2\}$$

represents the position of the outermost bp covered by a nucleosome, relative to its dyad. For a particle covering an odd number of bp, where the dyad position is well-defined, $\lfloor a/2 \rfloor = \frac{a-1}{2}$, which in the case of nucleosomes is $\lfloor a/2 \rfloor = 73$ bp. Note that $1 - \text{Occ}(i)$ is the probability that bp i is not covered by any nucleosome, *i.e.* the probability of finding linker DNA at bp i.

As we showed before (Chereji and Morozov, 2014), in the case of hard-core interactions alone, one can compute the single-particle distribution in an efficient way. Consider

$$n_1(i) = \frac{1}{Z} Z^-(i) e^{\beta[\mu - u(i)]} Z^+(i), \tag{2}$$

where $Z^-(i)$ and $Z^+(i)$ represent the partition functions for the domains to the left and to the right of the nucleosome with the dyad at position i. These two quantities satisfy the recursion relations:

$$Z^-(i) = Z^-(i-1) + Z^-(i-a) e^{\beta[\mu - u(i-a)]}, \tag{3}$$
$$Z^+(i) = Z^+(i+1) + Z^+(i+a) e^{\beta[\mu - u(i+a)]}. \tag{4}$$

Introducing new variables in log space

$$F(i) = \ln(Z^-(i)),$$
$$R(i) = \ln(Z^+(i)),$$

we obtain

$$F(i) = F(i-1) + \ln(1 + e^{F(i-a) - F(i-1) + \beta[\mu - u(i-a)]}), \tag{5}$$
$$R(i) = R(i+1) + \ln(1 + e^{R(i+a) - R(i+1) + \beta[\mu - u(i+a)]}). \tag{6}$$

These relations can be iterated starting from the boundary conditions:

$$F(i) = 0, \quad \forall i \leq a,$$
$$R(i) = 0, \quad \forall i \geq L - a.$$

From Eq. (2) the one-particle distribution becomes

$$n_1(i) = e^{F(i)+R(i)-\ln(Z)+\beta[\mu-u(i)]}, \tag{7}$$

where $\ln(Z) = F(L + \lfloor a/2 \rfloor + 1) = R(-\lfloor a/2 \rfloor)$. This system of equations [Eqs. (5), (6) and (7)] solves the forward problem, in which nucleosome formation and interaction energies are given and the distribution of particles is unknown.

Inverse Problem

In experiments such as MNase-seq, ChIP-seq, and ChIP-exo, genome-wide distributions of various DNA-binding proteins are determined, but the energetics of DNA binding are unknown. In order to predict protein-DNA binding and protein-protein interaction energies we need to solve the inverse problem, in which these energies are inferred starting from observed distributions of DNA-bound particles. In general, the inverse problem is much harder to solve compared to the corresponding forward problem.

Fortunately, it is straightforward to obtain nucleosome formation energies from the known distribution of nucleosomes, n_1(Locke et al., 2010; Chereji and Morozov, 2014).

From Eqs. (2), (3) and (4) we obtain

$$\frac{Z^-(i)}{Z^-(i-1)} = 1 + \frac{Zn_1(i-a)}{Z^-(i-1)Z^+(i-a)},$$
$$= 1 + \frac{n_1(i-a)}{\xi(i-a)}, \tag{8}$$

and

$$\frac{Z^+(i)}{Z^+(i+1)} = 1 + \frac{Zn_1(i+a)}{Z^-(i+a)Z^+(i+1)},$$
$$= 1 + \frac{n_1(i+a)}{\xi(i+1)}, \tag{9}$$

where

$$\xi(i) = \frac{Z^-(i+a-1)Z^+(i)}{Z}. \tag{10}$$

Note that a particle with the center at position i and a particle with the center at position $i+a-1$ overlap by exactly one bp. The overlapping bp is $i + \lfloor \frac{a}{2} \rfloor = i + a - 1 - \lfloor \frac{a}{2} \rfloor$. $Z^-(i+a-1)$ represents the partition function for the domain $[1, i + \lfloor \frac{a}{2} \rfloor)$, while $Z^+(i)$ represents the partition function for the domain $(i + \lfloor \frac{a}{2} \rfloor, L]$. Thus $Z^-(i+a-1)Z^+(i)$ represent all configurations which have bp $i + \lfloor \frac{a}{2} \rfloor$ unoccupied by a particle, so that

$$\xi(i) = 1 - \text{Occ}(i + \lfloor \frac{a}{2} \rfloor). \tag{11}$$

Using this, Eqs. (8) and (9) become

$$\frac{Z^-(i)}{Z^-(i-1)} = 1 + \frac{n_1(i-a)}{1-\mathrm{Occ}(i-a+\lfloor\frac{a}{2}\rfloor)}, \tag{12}$$

$$\frac{Z^+(i)}{Z^+(i+1)} = 1 + \frac{n_1(i+a)}{1-\mathrm{Occ}(i+1+\lfloor\frac{a}{2}\rfloor)}. \tag{13}$$

For $i \le a+\lfloor\frac{a}{2}\rfloor$, there is not enough space for a particle to completely fit in the domain $[1, i-\lfloor\frac{a}{2}\rfloor-1]$, so that

$$Z^-(i) = 1, \quad \forall i \le a + \lfloor\frac{a}{2}\rfloor. \tag{14}$$

For $i > a + \lfloor\frac{a}{2}\rfloor$ we have

$$\begin{aligned} Z^-(i) &= \frac{Z^-(i)}{Z^-(a+\lfloor\frac{a}{2}\rfloor)} \\ &= \frac{Z^-(i)}{\cancel{Z^-(i-1)}} \frac{\cancel{Z^-(i-1)}}{\cancel{Z^-(i-2)}} \cdots \frac{\cancel{Z^-(a+\lfloor\frac{a}{2}\rfloor+1)}}{Z^-(a+\lfloor\frac{a}{2}\rfloor)} \\ &= \prod_{k=a+\lfloor\frac{a}{2}\rfloor+1}^{i} \left[1 + \frac{n_1(k-a)}{1-\mathrm{Occ}(k-a+\lfloor\frac{a}{2}\rfloor)}\right] \\ &= \prod_{k=1+\lfloor\frac{a}{2}\rfloor+1}^{i-a} \left[1 + \frac{n_1(k)}{1-\mathrm{Occ}(k+\lfloor\frac{a}{2}\rfloor)}\right]. \end{aligned} \tag{15}$$

Similarly, for $i > L - a - \lfloor\frac{a}{2}\rfloor$, there is not enough space for a particle to fit in the domain $[i + \lfloor\frac{a}{2}\rfloor + 1, L]$, so that

$$Z^+(i) = 1, \quad \forall i > L - a - \lfloor\frac{a}{2}\rfloor. \tag{16}$$

For $i \le L - a - \lfloor\frac{a}{2}\rfloor$, we have

$$\begin{aligned} Z^+(i) &= \frac{Z^+(i)}{Z^+(L-a-\lfloor\frac{a}{2}\rfloor+1)} \\ &= \frac{Z^+(i)}{\cancel{Z^+(i+1)}} \frac{\cancel{Z^+(i+1)}}{\cancel{Z^+(i+2)}} \cdots \frac{\cancel{Z^+(L-a-\lfloor\frac{a}{2}\rfloor)}}{Z^+(L-a-\lfloor\frac{a}{2}\rfloor+1)} \\ &= \prod_{k=i}^{L-a-\lfloor\frac{a}{2}\rfloor} \left[1 + \frac{n_1(k+a)}{1-\mathrm{Occ}(k+1+\lfloor\frac{a}{2}\rfloor)}\right] \\ &= \prod_{k=i+a}^{L-\lfloor\frac{a}{2}\rfloor} \left[1 + \frac{n_1(k)}{1-\mathrm{Occ}(k-a+1+\lfloor\frac{a}{2}\rfloor)}\right] \\ &= \prod_{k=i+a}^{L-\lfloor\frac{a}{2}\rfloor} \left[1 + \frac{n_1(k)}{1-\mathrm{Occ}(k-\lfloor\frac{a}{2}\rfloor)}\right] \end{aligned} \tag{17}$$

Using Eqs. (14),(15),(16), and (17) we can compute the partition functions, and from Eq. (2) we obtain

$$e^{\beta[\mu-u(i)]} = \frac{Zn_1(i)}{Z^-(i)Z^+(i)},$$
$$\Rightarrow \beta[u(i)-\mu] = -\ln\left[\frac{Zn_1(i)}{Z^-(i)Z^+(i)}\right], \tag{18}$$

with $Z = Z^-(L+1+\lfloor\frac{a}{2}\rfloor) = Z^+(-\lfloor\frac{a}{2}\rfloor)$. The chemical potential μ can be determined from Eq. (2) if we know the average nucleosome density $< n_1(i) >_{i\in[1,L]}$. In practice, the real average nucleosome density is hard to estimate with accuracy, and we will use μ as a reference for all energies, and as a fitting parameter.

Energy models

We consider three models of nucleosome formation energies. The first model completely neglects DNA-sequence dependence of nucleosome formation and assumes that the only major energy contributions are given by external factors such as chromatin remodelers, which are responsible *e.g.* for generating the stereotypical nucleosome organization near the TSS of active genes. The second model is the "$N = 2$ position-independent model", introduced in Locke et al. (2010). Finally, the third model is a combination of the previous two.

Sequence-independent minimal model

For the sequence-independent model, we use the average nucleosome distribution at the TSS of active genes in order to infer the corresponding formation energy, $u^{\mathrm{SI}}(i)$, which can generate this distribution. We use Eq. (18) to obtain the effective energy potential generated by the action of all external factors. We assume that these factors contribute mostly near TSS, and their contribution vanishes away from TSS, so that we can eliminate the constant chemical potential from Eq. (18) by using the asymptotic value of the external potential

$$\lim_{i\to\infty} u^{\mathrm{SI}}(i) = 0 \Rightarrow \beta\mu = \lim_{i\to\infty} \beta[\mu - u^{\mathrm{SI}}(i)].$$

This way, we obtain the external potential shown in Figure 6A (black line), $u^{\mathrm{SI}}(i)$. This energy profile contains a potential barrier over the NDR and a potential well at the +1 nucleosome position. This suggests an anchoring mechanism that generates a strong bias for the location of +1 nucleosomes, which are known to be well-positioned *in vivo*.

To generate the genome-wide nucleosome distribution predicted by this model, we create an energy landscape which is flat except for 2 kb regions around the TSS of active genes, where the anchoring potential (black line in Figure 6A) is added.

Sequence-dependent model

The "$N = 2$ position-independent model" (Locke et al., 2010; Chereji and Morozov, 2011) assumes that the sequence-dependent part of the nucleosome formation energy, $u^{\mathrm{SD}}(i)$, corresponding to a nucleosome that occupies the DNA sequence between nucleotides $i - \lfloor\frac{a}{2}\rfloor$ and $i + \lfloor\frac{a}{2}\rfloor$, depends only

on the mono- and dinucleotide counts in the nucleosomal DNA:

$$u^{\mathrm{SD}}(i) = \sum_{k=i-\lfloor \frac{a}{2} \rfloor}^{i+\lfloor \frac{a}{2} \rfloor} \epsilon_{S_k/\widetilde{S}_k} + \sum_{k=i-\lfloor \frac{a}{2} \rfloor}^{i+\lfloor \frac{a}{2} \rfloor -1} \epsilon_{S_k S_{k+1}/\widetilde{S}_{k+1}\widetilde{S}_k}$$

where $\epsilon_{S_k/\widetilde{S}_k}$ is the energy contribution of the mononucleotide pair $S_k/\widetilde{S}_k$, and $\epsilon_{S_k S_{k+1}/\widetilde{S}_{k+1}\widetilde{S}_k}$ is the energy contribution of the dinucleotide pair $S_k S_{k+1}/\widetilde{S}_{k+1}\widetilde{S}_k$ ($\widetilde{S}_k$ is a nucleotide complementary to S_k). Because for each DNA sequence and its reverse complement the nucleosome formation energies are the same, $u^{SD}(i)$ is a function of only 12 unique parameters: $\epsilon_{A/T}$, $\epsilon_{C/G}$, $\epsilon_{AA/TT}$, $\epsilon_{AC/GT}$, $\epsilon_{AG/CT}$, $\epsilon_{AT/AT}$, $\epsilon_{CA/TG}$, $\epsilon_{CC/GG}$, $\epsilon_{CG/CG}$, $\epsilon_{GA/TC}$, $\epsilon_{GC/GC}$, and $\epsilon_{TA/TA}$.

The energies in Eq. (18) can then be written as

$$\begin{aligned} u^{\mathrm{SD}}(i) - \mu &= \sum_{k=i-\lfloor \frac{a}{2} \rfloor}^{i+\lfloor \frac{a}{2} \rfloor} \epsilon_{S_k} + \sum_{k=i-\lfloor \frac{a}{2} \rfloor}^{i+\lfloor \frac{a}{2} \rfloor -1} \epsilon_{S_k S_{k+1}} - \mu \\ &= \begin{pmatrix} m^i_{A/T} & m^i_{C/G} & m^i_{AA/TT} & \cdots & m^i_{TA/TA} & -1 \end{pmatrix} \begin{pmatrix} \epsilon_{A/T} \\ \vdots \\ \epsilon_{TA/TA} \\ \mu \end{pmatrix}, \end{aligned} \tag{19}$$

where μ is the chemical potential and $m^i_{X/\widetilde{X}}$, $m^i_{XY/\widetilde{Y}\widetilde{X}}$ are the counts of mono- and dinucleotide pairs $X/\widetilde{X}$ and $XY/\widetilde{Y}\widetilde{X}$ in the DNA sequence containing nucleotides between $i - \lfloor \frac{a}{2} \rfloor$ and $i + \lfloor \frac{a}{2} \rfloor$, respectively.

For all dyad positions, i, we obtain a system of P equations

$$E - \mu \;=\; M \begin{pmatrix} \epsilon \\ \mu \end{pmatrix}, \tag{20}$$

where $E - \mu$ is a column vector of dimension P, each row containing one element $u^{\mathrm{SD}}(i) - \mu$ from Eq. (19). $\begin{pmatrix} \epsilon \\ \mu \end{pmatrix}$ is the column vector of fitting parameters from Eq. (19), and M is a $P \times 13$ matrix with mono- and dinucleotide counts, and -1's in the last column. Using Eqs. (20), we obtain the energy parameters ϵ and μ by a least-squares fit.

Because in every DNA sequence the number of mononucleotides is equal to the length of the sequence and the number of dinucleotides is equal to the length of the sequence minus one, the columns of the matrix M are not linearly independent: two columns can be expressed as a linear combination of the others. To make the set of fitted parameters unique, we impose the following constraints

$$\begin{aligned} \epsilon_{A/T} + \epsilon_{C/G} &= 0, \\ \epsilon_{AA/TT} + \epsilon_{AC/GT} + \ldots + \epsilon_{TA/TA} &= 0, \end{aligned}$$

as explained in Chereji and Morozov (2011).

Full model

We also construct a third model in which the full nucleosome formation energy is the sum of the sequence-independent energy, u^{SI}, and the sequence-dependent energy, u^{SD}. After fitting the "$N = 2$ position-independent model" described above, we add u^{SI} and u^{SD}. The results of all three models considered above are shown in Supplemental Figure S8.

Differential MNase-seq

MNase digestion of chromatin of *Drosophila* embryos was performed as previously described (Petesch and Lis, 2008), with several modifications. Briefly, 2 g of embryos collected overnight were homogenized in 5 ml of buffer A1 (60mM KCl, 15 mM $NaCl_2$, 4mM $MgCl_2$, 15 mM HEPES pH 7.6, 0.5 mM DTT, 0.5% Triton X-100, protease inhibitors) containing 2% of formaldehyde, and crosslinked for 15' at room temperature. Reaction was stopped by adding 1M of glycine to a final concentration of 225 mM followed by 5' incubation. Cross-linked nuclei were filtered through Miracloth (475855, EMD Millipore), loaded on top of the sucrose cushion (0.3 M sucrose in buffer A1) and centrifuged at 700 g, 4°C for 5'. Nuclei were washed 2X in buffer A1, 2X in buffer D (25% glycerol, 5 mM Mg Acetate, 50 mM Tris pH 8.0, 0.1 mM EDTA, 5 mM DTT) and re-suspended in 1 ml of buffer MN (60 mM KCl, 15 mM NaCl, 15 mM Tris pH 7.4, 0.5 mM DTT, 0.25 M sucrose, 1.0 mM $CaCl_2$).

Micrococcal nuclease (MNase) digestion of chromatin was performed by the addition of 10 ($MNase^{LOW}$) to 250 ($MNase^{HIGH}$) total units of MNase (70196Y, Affymetrix) to 200 μl of isolated nuclei. Following 10' of incubation at 25°C, the reactions were stopped with EDTA and SDS added to final concentrations of 12.5 mM and 0.5% respectively. MNase-digested DNA was de-crosslinked by incubation in elution buffer containing 1% SDS, 0.5 mg/ml Proteinase K, 0.1 M $NaHCO_3$ for 2 h at 37°C and 16 h at 65°C, purified with QIAquick PCR Purification kit (28106, QIAgen), and analysed on 2% agarose gel. DNA fragments corresponding to mononucleosome (~147 bp) were excised from the gel and purified for further analysis.

A similar protocol was used for MNase digestion of chromatin from $\sim 2 \times 10^8$ *Drosophila* S2 cells. For S2 cells, DNA fragments corresponding to mono- (~147 bp) and dinucleosomes (~300-350 bp) were isolated on a gel. Libraries for Illumina sequencing were prepared from 10 ng of DNA by Service XS (Netherlands) with Illumina kits, or in-house with SureSelect kit (G9691A, Agilent) starting from the end-repair step. For the input controls, libraries from sonicated or MNase-digested genomic DNA were prepared. Library quality was assessed on Bioanalyzer using the DNA1000 kit (5067-1504, Agilent). Libraries were paired-end sequenced (2×25 bp or 2×50 bp) on Illumina HiSeq 2000.

Differential MNase-ChIP-seq

For $MNase^{HIGH}$-ChIP-seq, 1 ml of nuclei was prepared from 2 g of *Drosophila* embryos as described above, followed by the complete digestion of chromatin to mononucleosomes with 250 U of MNase per 200 μl of nuclei for 10' at 25°C. After that, nuclei were immediately lysed for 10' at 4°C by the addition of equal volume of 2X ChIP lysis buffer (2% SDS, 20 mM EDTA, 100 mM Tris-HCl pH 8.1, 0.2 mM PMSF), and stored at -80°C before use.

For $MNase^{LOW}$-ChIP-seq, 12 ml of nuclei were prepared from 50 g of *Drosophila* embryos, followed by the digestion of chromatin with 10 U of MNase per 200 μl of nuclei for 10' at 25°C. After the reaction was stopped, nuclei were collected by centrifugation at 9000 g for 10' at 4°C and lysed for 1 hour at 4°C in 6 ml of a buffer containing 0.5 M NaCl, 10 mM HEPES pH 7.6, 1 mM EDTA and 0.2 mM of protease inhibitor PMSF. Lysed nuclei were spun at 15000 g for 15' at 4°C and supernatant containing soluble chromatin was loaded onto the 5-30% sucrose gradient prepared on Gradient Master (Biocomp). Molecular weight fractions were formed by centrifugation at 26000 rpm for 16 h at 4°C in SW 40 Ti swing rotor. 1 ml fractions were collected and 200 μl aliquots were de-crosslinked and analysed on a 2% agarose gel. Fractions containing mononucleosomes were used for chromatin immune-precipitation (ChIP).

ChIPs were performed as previously described (Moshkin et al., 2012). In brief, lysed nuclei from MNase^{HIGH} digestion or isolated mononucleosomes from MNase^{LOW} digestion experiments were diluted with 10 volumes of ChIP dilution buffer (1% Triton X-100, 150 mM NaCl, 2 mM EDTA, 20mM Tris-HCl pH 8.1, 0.2 mM PMSF), incubated with ChIP-grade antibodies against histone H3 (ab1791, Abcam) or H2B (Moshkin et al., 2012) overnight at 4°C, and precipitated with pre-blocked protein A agarose (16-157, EMD Millipore). Following extensive washes: 3 times with a buffer containing 0.1% SDS, 1% Triton X-100, 2 mM EDTA, 20 mM Tris-HCl pH 8.1 and 150 mM NaCl and once with a similar buffer containing 500 mM NaCl, DNA was de-crosslinked and purified with QIAquick PCR Purification kit (28106, QIAgen). DNA libraries for Illumina sequencing were prepared as above.

Supplemental Figures

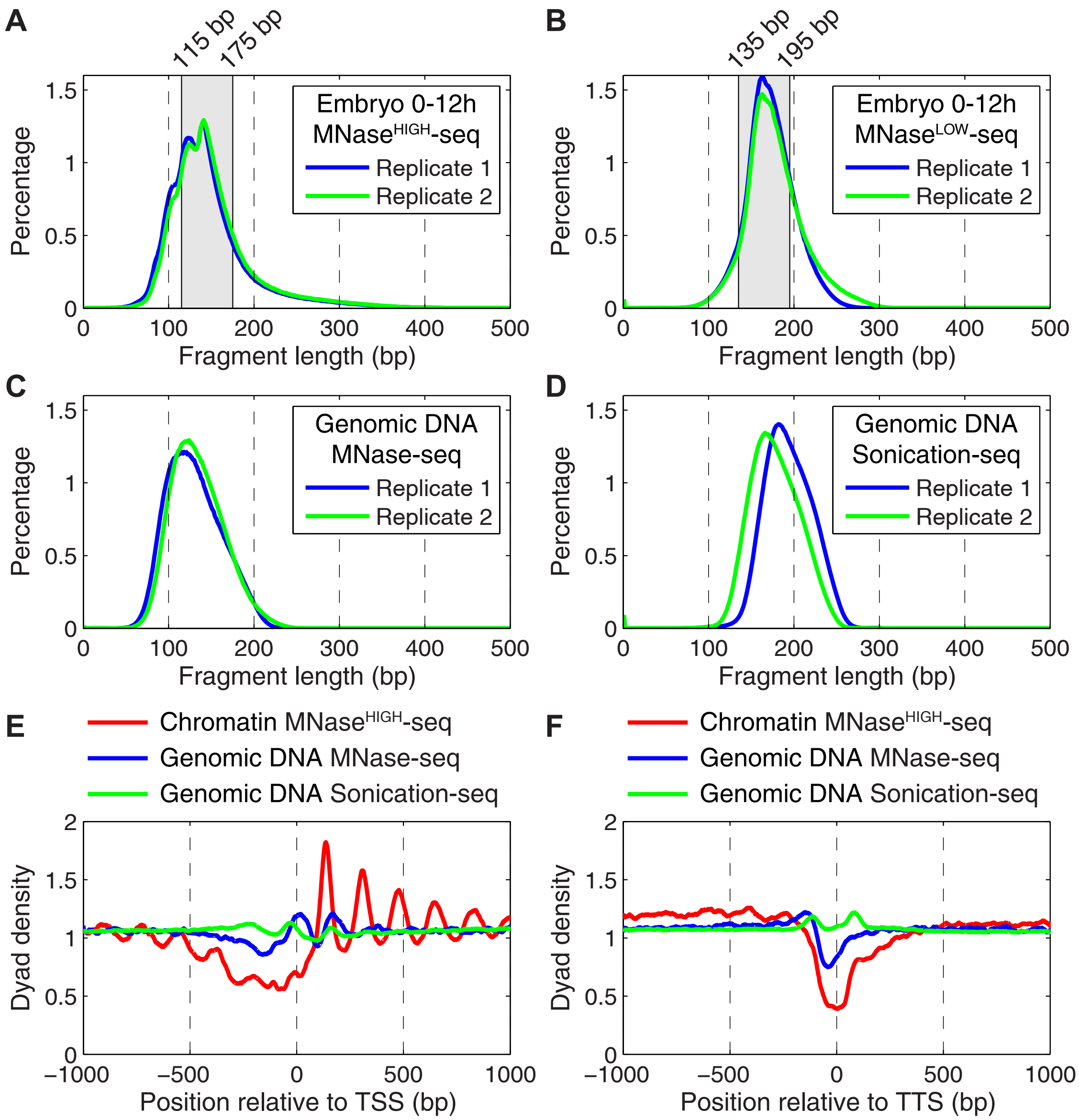

Figure S1. Length distributions of sequenced DNA fragments in MNase-seq and control experiments, and nucleosome distributions in the vicinity of TSS and TTS. (A,B) Length distribution of mononucleosomal MNase^{HIGH} (A) and MNase^{LOW} (B) DNA fragment lengths prepared from Drosophila embryos (Figure 1B), as determined by paired-end sequencing (MNase-seq). Consistently, average fragment lengths are ~145 bp and ~165 bp for MNase^{HIGH}–seq and MNase^{LOW}–seq, respectively. For further analysis fragments of 145 ± 30 bp and 165 ± 30 bp were retained in MNase^{HIGH} and MNase^{LOW} experiments, respectively (grey bars). (C) Fragment length distribution of genomic (nucleosome-free) DNA digested with MNase. (D) Fragment length distribution of sonicated genomic DNA. For each experiment in (A-D), two independent biological replicates were analyzed. (E,F) Averaged plots of MNase^{HIGH} (red curve) normalized nucleosome dyad profiles in comparison to dyad profiles of MNase-digested (blue curve) and sonicated (green curve) genomic DNA aligned at TSS (E) and TTS (F). Note that all fragment lengths were retained in genomic control experiments.

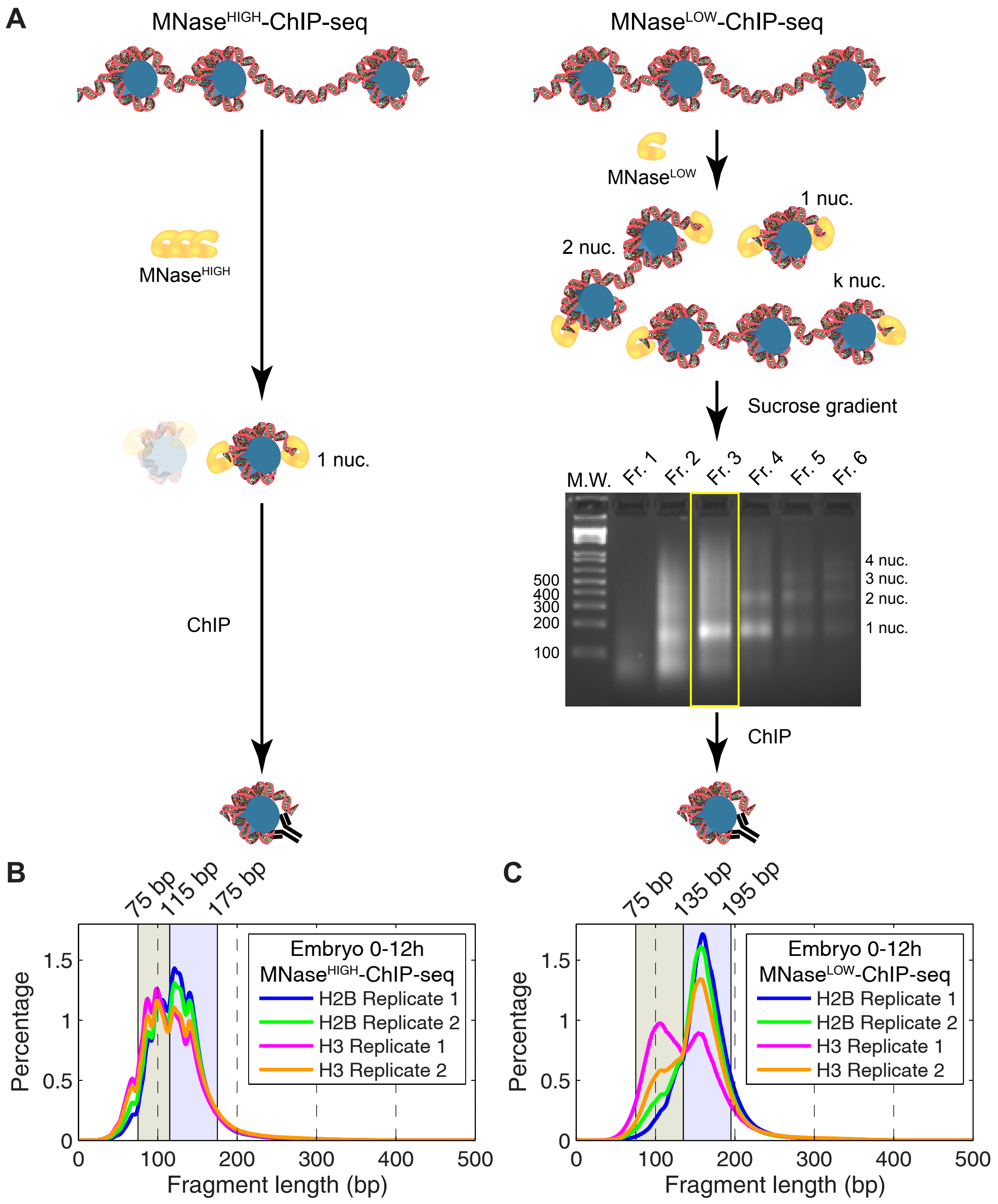

Figure S2. MNase digestion of *Drosophila* embryo chromatin followed by immunoprecipitation (MNase-ChIP-seq). (A) An outline of MNase-ChIP-seq nucleosome profiling. As in Figure 1A, chromatin of 0-12 hour Drosophila embryos was partially or completely digested with low and high concentrations of MNase (MNase^{LOW} and MNase^{HIGH}, respectively). Mononucleosomes obtained from the complete digestion (MNase^{HIGH}) were precipitated with anti-H2B or anti-H3 antibodies, and the resulting DNA fragments were sequenced using paired-end reads (MNase^{HIGH}-ChIP-seq, left panel). Mono- and oligonucleosomes obtained from the partial digestion (MNase^{LOW}) were first separated according to molecular weight by sucrose gradient fractionation. The resulting fractions were analysed on an agarose gel, and the fraction containing mononucleosomes was used for ChIP-seq with anti-H2B or anti-H3 antibodies (MNase^{LOW}-ChIP-seq, right panel). (B,C) Length distributions of mononucleosomal MNase^{HIGH}-ChIP-seq (B) and MNase^{LOW}-ChIP-seq (C) DNA fragments, as determined by paired-end sequencing. For each experiment, two independent biological replicates were analyzed. A sizable number of fragments in MNase^{HIGH}-ChIP-seq and MNase^{LOW}-ChIP-seq experiments were shorter than 147 bp, suggesting that these nucleosomes were either unwrapped or partially disassembled. For further analysis we used fragments of 145 ± 30 bp (MNase^{HIGH}-ChIP-seq) and 165 ± 30 bp (MNase^{LOW}-ChIP-seq), as in Figure S1 (lighter grey bars). In addition, we considered shorter fragments of 95 ± 20 bp (MNase^{HIGH}-ChIP-seq) and 105 ± 30 bp (MNase^{LOW}-ChIP-seq) in H3 experiments (darker grey bars).

A

		Embryo 0-12h								S2 cells			
		MNase^{HIGH} 1n	MNase^{HIGH}-ChIP$^{H2B}_{115-175}$	MNase^{HIGH}-ChIP$^{H3}_{115-175}$	MNase^{HIGH}-ChIP$^{H3}_{75-115}$	MNase^{LOW} 1n	MNase^{LOW}-ChIP$^{H2B}_{115-175}$	MNase^{LOW}-ChIP$^{H3}_{115-175}$	MNase^{LOW}-ChIP$^{H3}_{75-115}$	MNase^{HIGH} 1n	MNase^{LOW} 1n	MNase^{LOW} 2n	MNase^{LOW} 1n+2n
Embryo 0-12h	MNase^{HIGH} 1n	1.00	0.74	0.80	0.38	0.09	0.06	-0.19	-0.23	0.67	0.16	0.44	0.29
	MNase^{HIGH}-ChIP$^{H2B}_{115-175}$	0.74	1.00	0.84	0.55	0.25	0.28	0.01	-0.04	0.54	0.25	0.42	0.35
	MNase^{HIGH}-ChIP$^{H3}_{115-175}$	0.80	0.84	1.00	0.48	0.12	0.12	-0.12	-0.17	0.61	0.17	0.41	0.28
	MNase^{HIGH}-ChIP$^{H3}_{75-115}$	0.38	0.55	0.48	1.00	0.35	0.43	0.26	0.27	0.20	0.32	0.23	0.33
	MNase^{LOW} 1n	0.09	0.25	0.12	0.35	1.00	0.76	0.67	0.56	0.02	0.56	0.19	0.49
	MNase^{LOW}-ChIP$^{H2B}_{115-175}$	0.06	0.28	0.12	0.43	0.76	1.00	0.76	0.61	-0.05	0.53	0.14	0.45
	MNase^{LOW}-ChIP$^{H3}_{115-175}$	-0.19	0.01	-0.12	0.26	0.67	0.76	1.00	0.71	-0.25	0.41	-0.02	0.29
	MNase^{LOW}-ChIP$^{H3}_{75-115}$	-0.23	-0.04	-0.17	0.27	0.56	0.61	0.71	1.00	-0.27	0.36	-0.05	0.25
S2 cells	MNase^{HIGH} 1n	0.67	0.54	0.61	0.20	0.02	-0.05	-0.25	-0.27	1.00	0.39	0.69	0.55
	MNase^{LOW} 1n	0.16	0.25	0.17	0.32	0.56	0.53	0.41	0.36	0.39	1.00	0.53	0.95
	MNase^{LOW} 2n	0.44	0.42	0.41	0.23	0.19	0.14	-0.02	-0.05	0.69	0.53	1.00	0.78
	MNase^{LOW} 1n+2n	0.29	0.35	0.28	0.33	0.49	0.45	0.29	0.25	0.55	0.95	0.78	1.00

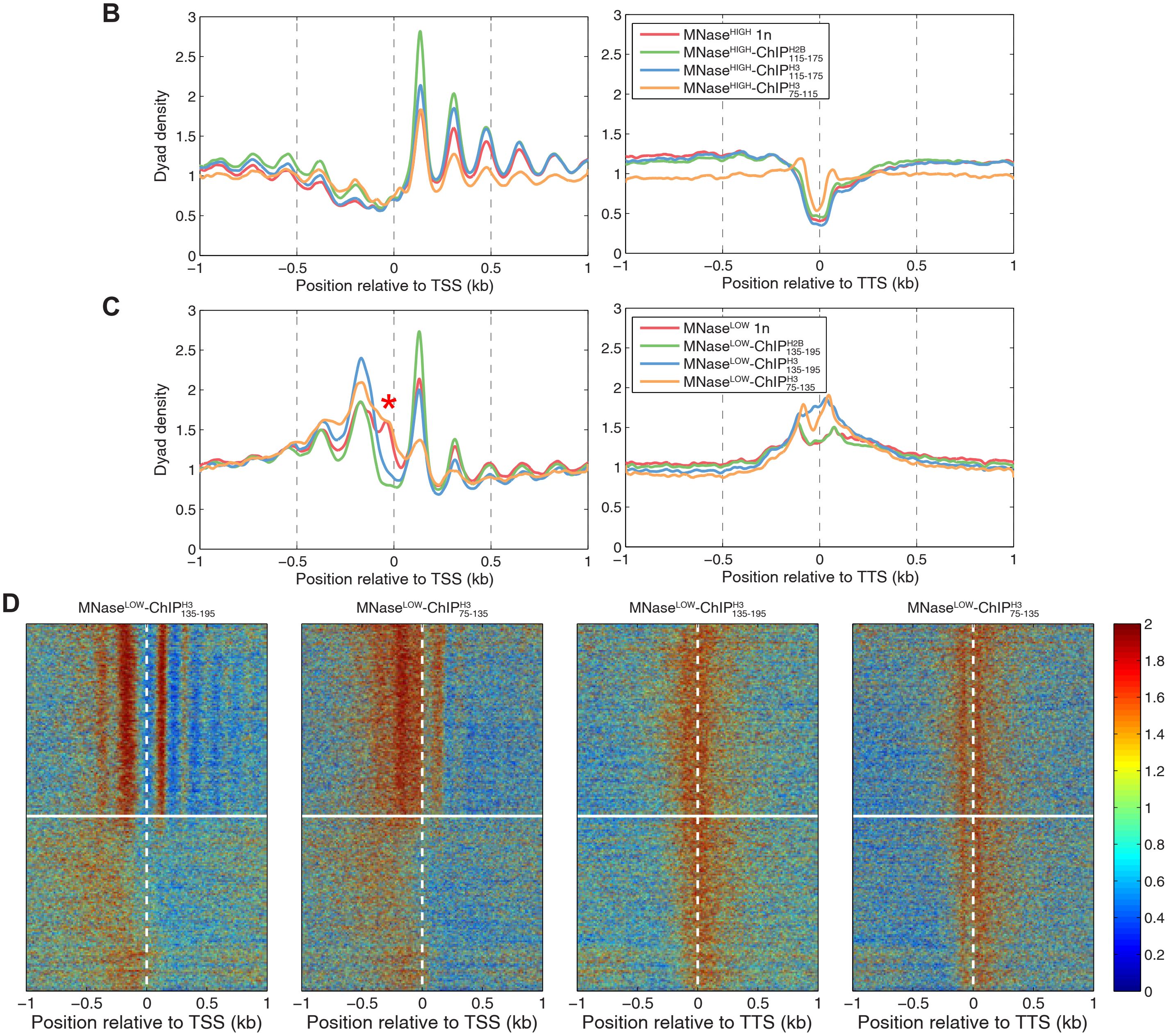

Figure S3. Caption on the next page.

Figure S3. Distributions of nucleosomes identified by MNase-ChIP-seq in the vicinity of TSS and TTS, and genome-wide correlations between nucleosome occupancy profiles. (A) Correlations between genome-wide nucleosome occupancy profiles for *Drosophila* embryos and S2 cells. Occupancy profiles derived from $\text{MNase}^{\text{HIGH}}$ and $\text{MNase}^{\text{LOW}}$-ChIP-seq with anti-H2B or anti-H3 antibodies for *Drosophila* embryos are labelled $\text{MNase}^{\text{HIGH}}$-$\text{ChIP}^{\text{H2B}}$, $\text{MNase}^{\text{HIGH}}$-$\text{ChIP}^{\text{H3}}$, $\text{MNase}^{\text{LOW}}$-$\text{ChIP}^{\text{H2B}}$, and $\text{MNase}^{\text{LOW}}$-$\text{ChIP}^{\text{H3}}$ (subscripts indicate the range of DNA fragment lengths used in creating each profile). Occupancy profiles determined by MNase-seq are labelled $\text{MNase}^{\text{HIGH}}$ and $\text{MNase}^{\text{LOW}}$; 1n and 2n indicate mono- and dinucleosomes, respectively. (B) Averaged plots of $\text{MNase}^{\text{HIGH}}$-seq and $\text{MNase}^{\text{HIGH}}$-ChIP-seq nucleosome dyad profiles aligned at TSS (left panel) and TTS (right panel). Nucleosome profiles are labelled as in (A). (C) Averaged plots of $\text{MNase}^{\text{LOW}}$-seq and $\text{MNase}^{\text{LOW}}$-ChIP-seq nucleosome dyad profiles aligned at TSS (left panel) and TTS (right panel). Nucleosome profiles are labelled as in (A). (D) Heat maps of $\text{MNase}^{\text{LOW}}$-$\text{ChIP}^{\text{H3}}$-seq normalized nucleosome dyad profiles around TSS and TTS for long (165 ± 30 bp) and short (115 ± 30 bp) DNA fragments. Genes are ordered by their expression levels from high (top) to low (bottom) using RNA-seq data for Drosophila embryos from modENCODE (www.modencode.org) (Gerstein et al., 2014). Horizontal white lines separate active and silent genes.

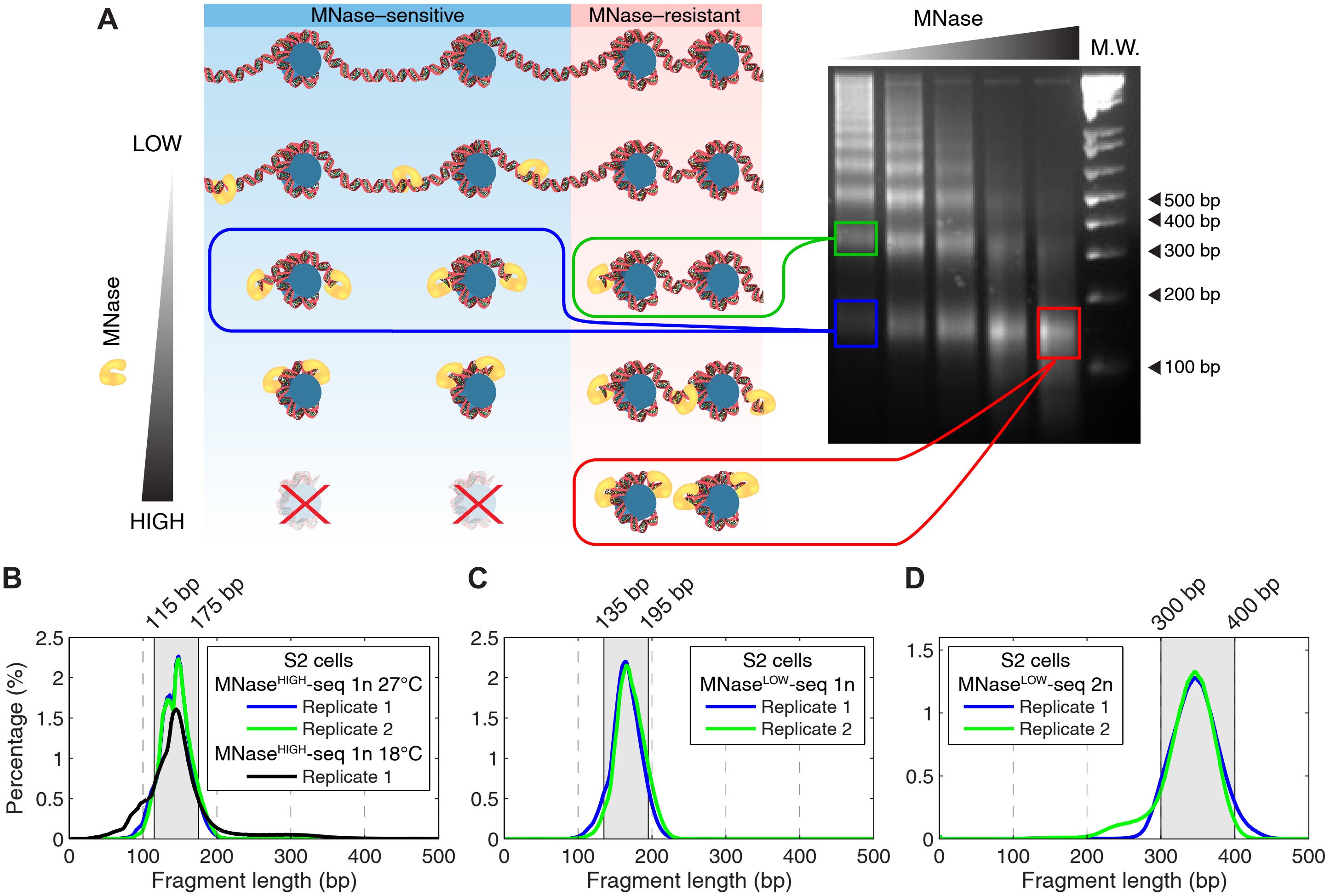

Figure S4. Mapping of nucleosome positions in *Drosophila* S2 cells. (A) The nucleosome profile at low MNase concentration is reconstructed by sequencing both mono- and di-nucleosomal DNA fragments (blue and green rectangles on the agarose gel). The nucleosome profile at high MNase is based on mononucleosomal DNA fragments (red rectangle on the agarose gel). (B-D) Distributions of mononucleosomal DNA fragment lengths at high MNase concentration (B; MNase^{HIGH} 1n at 18°C and 27°C), low MNase concentration (C; MNase^{LOW} 1n at 27°C), and of dinucleosomal DNA fragment lengths at low MNase concentration (D; ^{LOW} 2n at 27°C). For each experiment except MNase^{HIGH} 1n at 18°C, two independent biological replicates were analysed, and the data from both replicates was combined for further analysis. The average fragment lengths were ∼145 bp and ∼165 bp in MNase^{HIGH}-seq and MNase^{LOW}-seq experiments respectively, and ∼345 bp with dinucleosomal DNA. For further analysis, DNA fragments of 145 ± 30 bp were selected for MNase^{HIGH} 1n, 165 ± 30 bp for MNase^{LOW} 1n, and 350 ± 50 bp for MNase^{LOW} 2n (grey bars).

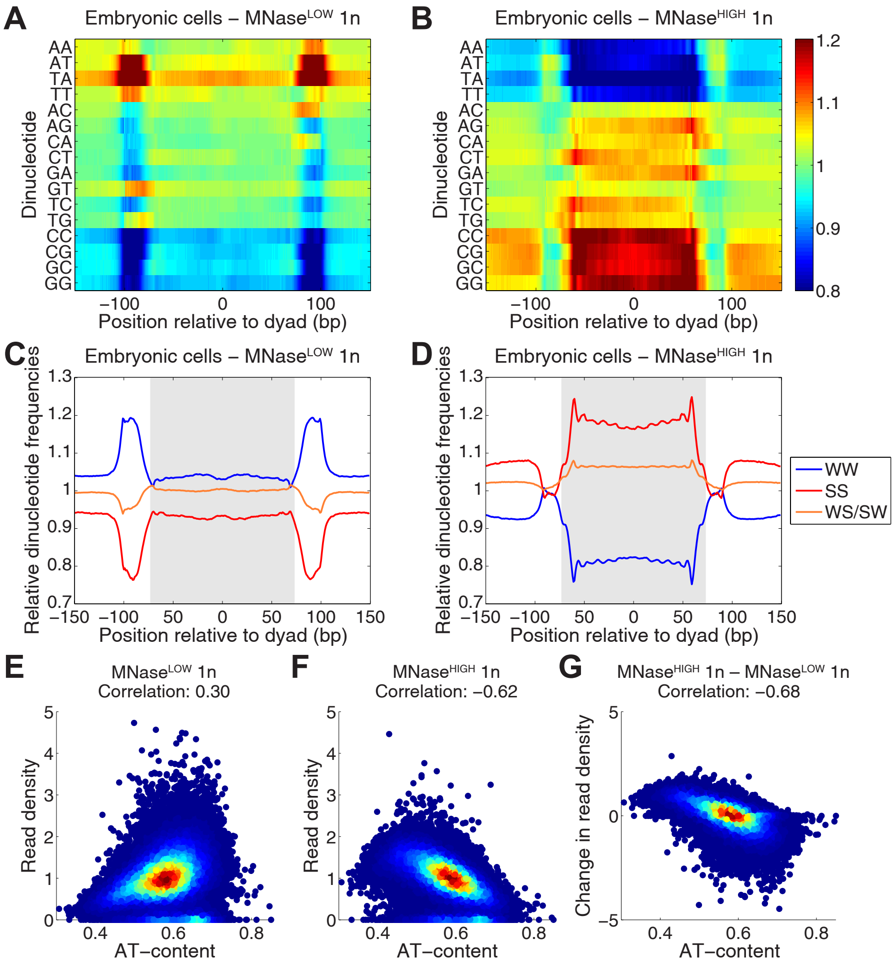

Figure S5. MNase-sensitive and MNase-resistant nucleosomes are associated with distinct sequence motifs. (A-G) Same as (A-G) in Figure 3 but for 0-12 h embryonic cells.

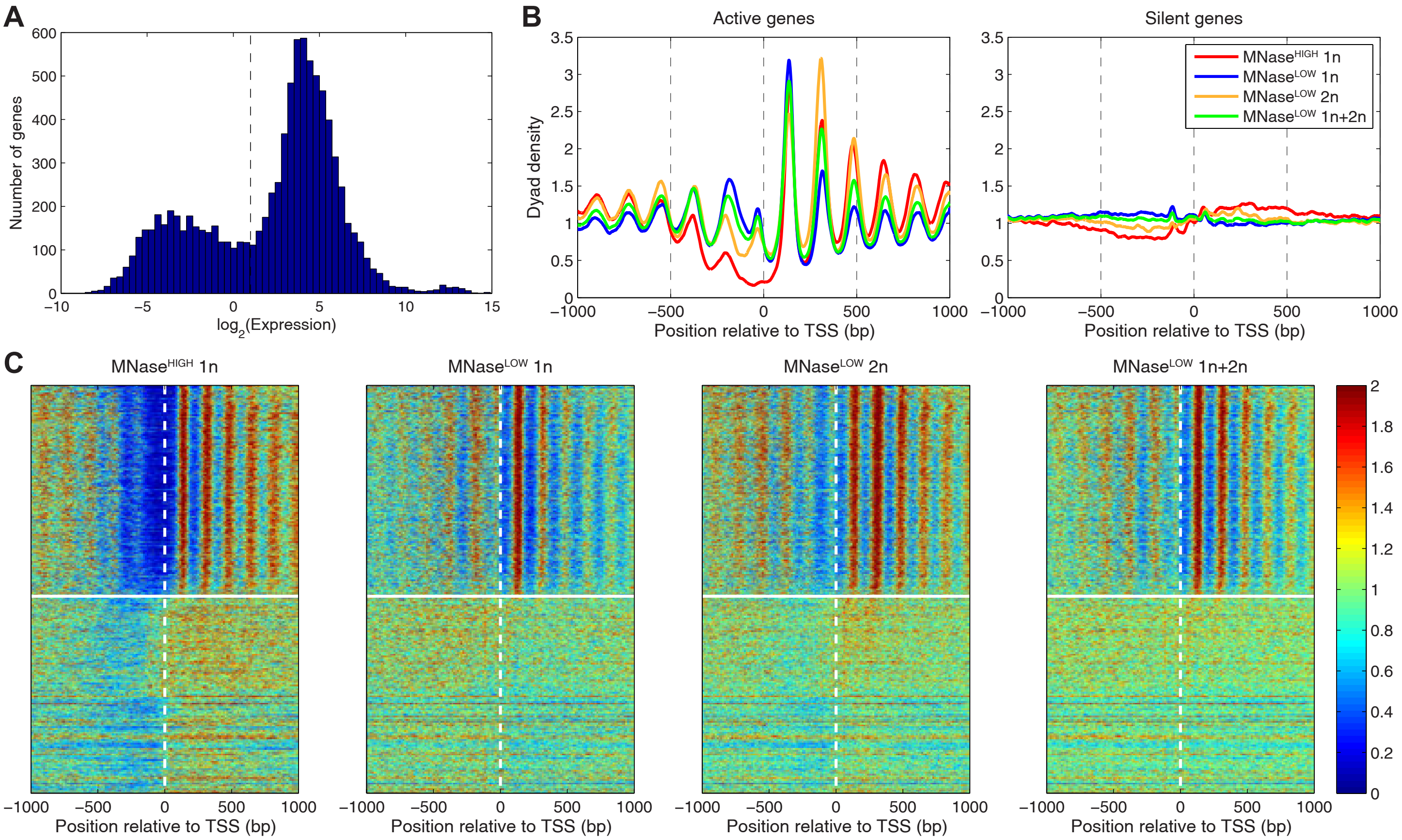

Figure S6. Nucleosome organization around coding regions in *Drosophila* S2 cells follows bimodality of gene expression levels. (A) Gene expression levels (RNA-seq data for Drosophila S2 cells, Methods) show a bimodal distribution: active and silent genes are separated by a dashed vertical line. (B) Averaged plots of MNase^{HIGH} 1n, MNase^{LOW} 1n, MNase^{LOW} 2n and MNase^{LOW} 1n+2n normalized nucleosome dyad profiles aligned at TSS, for active and silent genes. (C) Heat maps of MNase^{HIGH} 1n, MNase^{LOW} 1n, MNase^{LOW} 2n and MNase^{LOW} 1n+2n normalized nucleosome dyad profiles around TSS. Genes were ordered by their expression levels from high (top) to low (bottom) as in (A). Vertical dashed lines mark TSS and TTS positions; horizontal lines mark the boundary between active and silent genes.

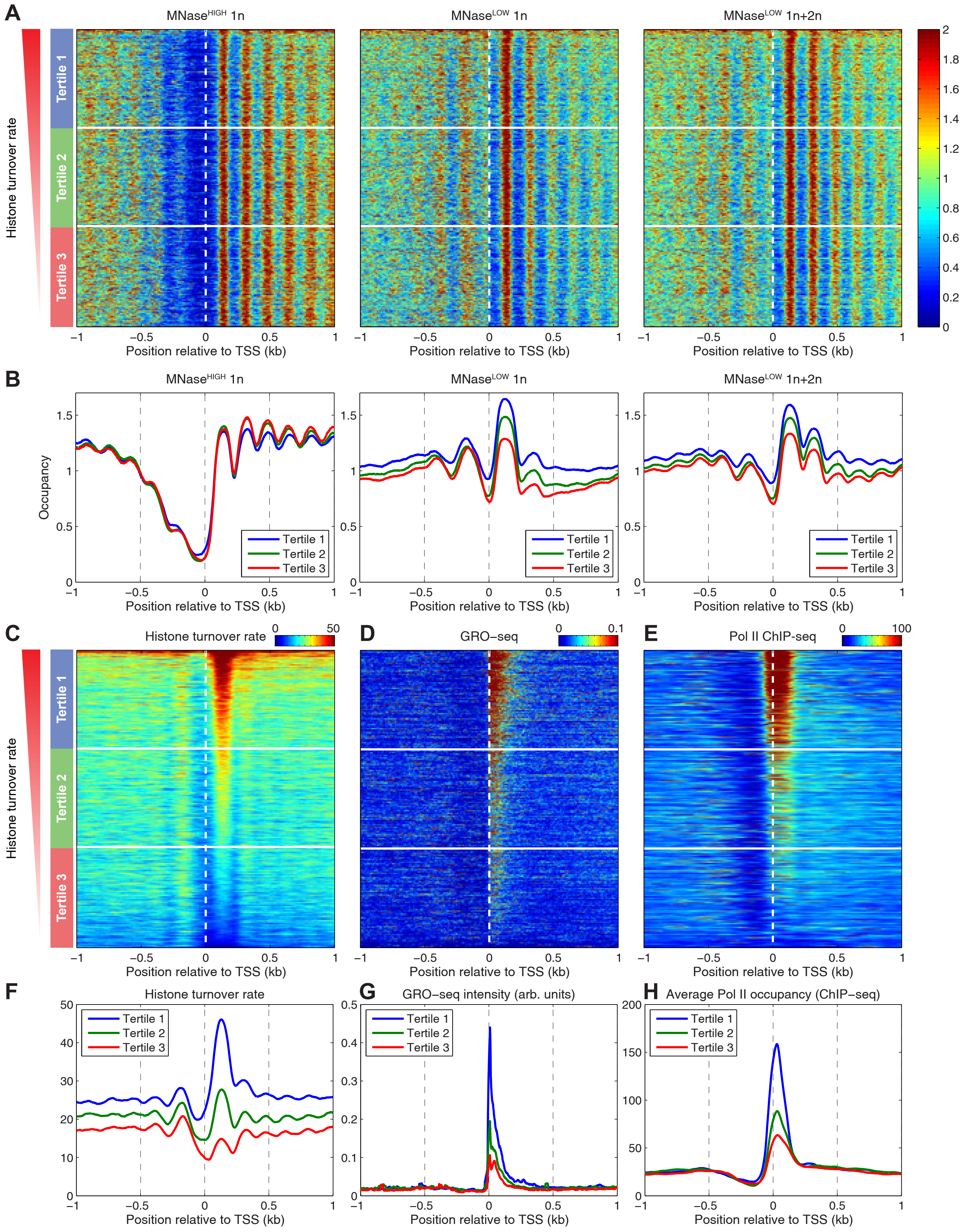

Figure S7. Caption on the next page.

Figure S7. Nucleosome organization and histone turnover rates. (A) Heat maps of MNase^{HIGH} 1n, MNase^{LOW} 1n, MNase^{LOW} 2n and MNase^{LOW} 1n+2n normalized nucleosome dyad profiles around TSS, for active genes sorted by histone turnover rates (Teves and Henikoff, 2014). Active genes in *Drosophila* S2 cells (Figure S6A), which exhibit nucleosome phasing around TSS, were sorted according to the average histone turnover rates in the [TSS, TSS+250] region (Teves and Henikoff, 2014) (histone turnover rates decrease from top to bottom), and split into three equal-size tertiles. (B) Averaged plots of MNase^{HIGH} 1n, MNase^{LOW} 1n, MNase^{LOW} 2n and MNase^{LOW} 1n+2n normalized nucleosome occupancy profiles aligned at TSS, for the three tertiles from (A). (C) Heat map of histone turnover rates (Teves and Henikoff, 2014) in the vicinity of TSS. (D,E) Heat maps of Pol II binding around TSS identified by GRO-seq (Core et al., 2012) (D; polymerase positions plotted) and ChIP-seq (Core et al., 2012) (E; fragment coverage plotted), for active genes sorted as in (A). (F) Average histone turnover rates for the three tertiles from (A). (G,H) Averaged plots of Pol II binding aligned by TSS, based on GRO-seq (G) and ChIP-seq (H) data, for the three tertiles from (A).

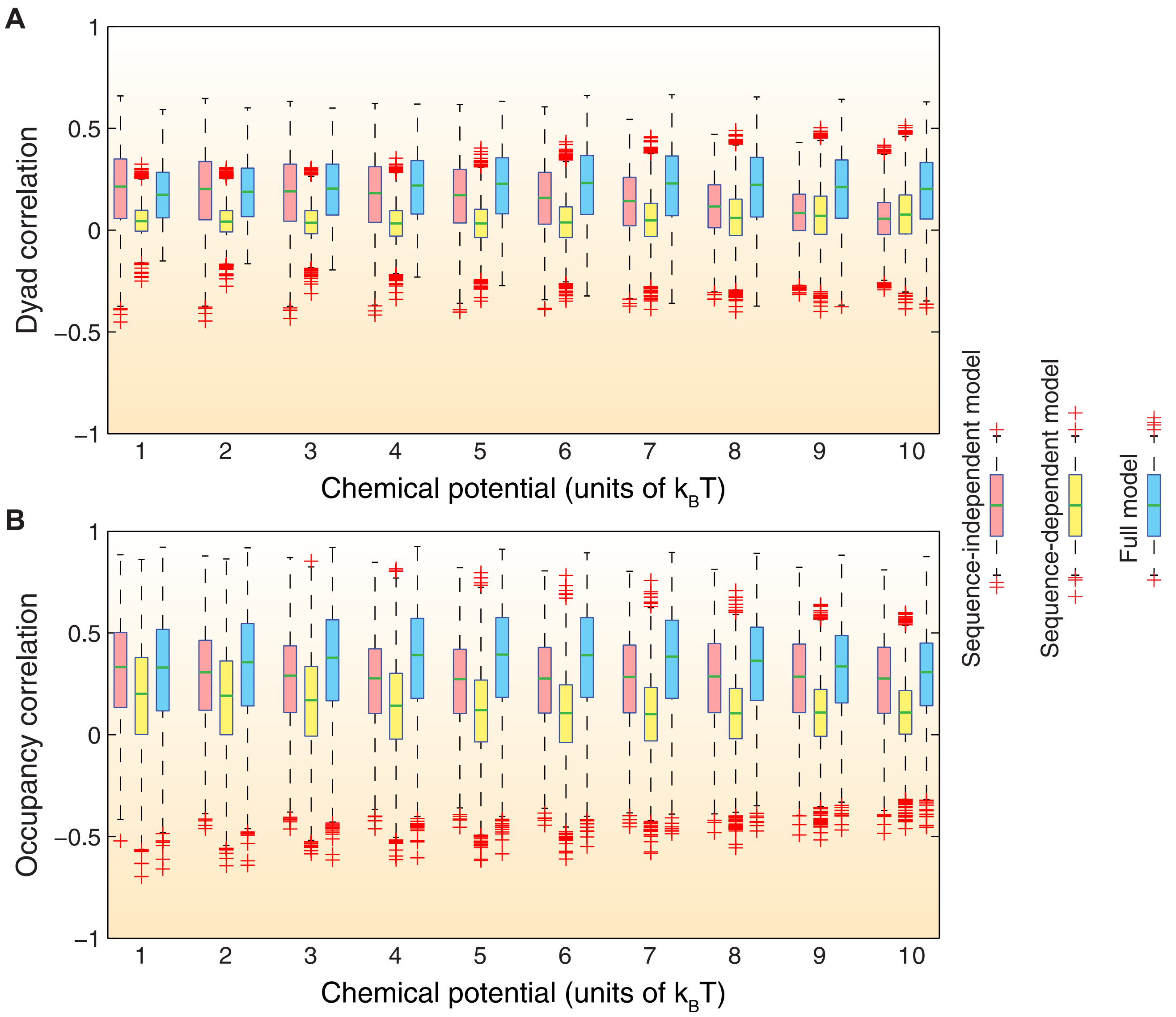

Figure S8. Including external nucleosome anchoring potential improves predictive power of the model. Box plots of Pearson's correlation coefficients obtained by considering genomic regions with phased arrays of nucleosomes ([TSS-500, TSS+1000] for active genes) in *Drosophila* S2 cells. (A) Correlations between predicted and observed dyad density profiles for a range of chemical potentials (histone octamer concentrations). (B) Correlations between predicted and observed nucleosome occupancy profiles. Predictions were made using a sequence-dependent model, a sequence-independent model (black line in Figure 6A), and a full model in which the sequence-dependent and sequence-independent contributions are combined (Supplemental Methods). Box plots indicate median correlation (green lines), the 25th and 75th percentiles (boxes), the range of the correlation coefficients not considered outliers (black dashed lines), and outliers (red crosses). Data points are considered outliers if they are larger than $q_3 + 1.5(q_3 - q_1)$ or smaller than $q_1 - 1.5(q_3 - q_1)$, where q_1 and q_3 are the 25th and 75th percentiles, respectively.

Supplemental Tables

Table S1. Summary of paired-end sequencing data. Occupancy correlation refers to the genome-wide linear correlation coefficient between Replicate 1 and Replicate 2 nucleosome occupancy profiles.

Cell type	Sample	Number of reads		Occupancy correlation
		Replicate 1	Replicate 2	
Embryo 0-12h	$\mathrm{MNase}^{\mathrm{HIGH}}$ 1n	7,225,834	5,216,329	0.74
	$\mathrm{MNase}^{\mathrm{HIGH}}$-$\mathrm{ChIP}^{\mathrm{H2B}}_{115-175}$	11,403,635	7,418,349	0.80
	$\mathrm{MNase}^{\mathrm{HIGH}}$-$\mathrm{ChIP}^{\mathrm{H3}}_{115-175}$	7,454,787	5,999,965	0.78
	$\mathrm{MNase}^{\mathrm{HIGH}}$-$\mathrm{ChIP}^{\mathrm{H3}}_{75-115}$	7,590,548	5,139,287	0.67
	$\mathrm{MNase}^{\mathrm{LOW}}$ 1n	8,904,331	5,270,102	0.73
	$\mathrm{MNase}^{\mathrm{LOW}}$-$\mathrm{ChIP}^{\mathrm{H2B}}_{115-175}$	6,979,970	5,270,652	0.72
	$\mathrm{MNase}^{\mathrm{LOW}}$-$\mathrm{ChIP}^{\mathrm{H3}}_{115-175}$	2,227,814	4,830,926	0.67
	$\mathrm{MNase}^{\mathrm{LOW}}$-$\mathrm{ChIP}^{\mathrm{H3}}_{75-115}$	2,605,103	2,575,409	0.53
S2 cells	$\mathrm{MNase}^{\mathrm{HIGH}}$ 1n	48,999,334	35,595,140	0.94
	$\mathrm{MNase}^{\mathrm{LOW}}$ 1n	54,222,987	21,435,444	0.92
	$\mathrm{MNase}^{\mathrm{LOW}}$ 2n	12,372,555	11,444,587	0.78
S2 cells in 18 °C	$\mathrm{MNase}^{\mathrm{HIGH}}$ 1n	3,435,272	–	–

Supplemental References

Chereji RV and Morozov AV. 2011. Statistical mechanics of nucleosomes constrained by higher-order chromatin structure. *J. Stat. Phys.* **144**: 379–404.

Chereji RV and Morozov AV. 2014. Ubiquitous nucleosome crowding in the yeast genome. *Proc. Natl. Acad. Sci. USA* **111**: 5236–5241.

Chereji RV, Tolkunov D, Locke G, and Morozov AV. 2011. Statistical mechanics of nucleosome ordering by chromatin-structure-induced two-body interactions. *Phys. Rev. E* **83**: 050903.

Chevereau G, Palmeira L, Thermes C, Arneodo A, and Vaillant C. 2009. Thermodynamics of intragenic nucleosome ordering. *Phys. Rev. Lett.* **103**: 188103.

Core LJ, Waterfall JJ, Gilchrist DA, Fargo DC, Kwak H, Adelman K, and Lis JT. 2012. Defining the status of RNA polymerase at promoters. *Cell Rep.* **2**: 1025–1035.

Gerstein MB, Rozowsky J, Yan KK, Wang D, Cheng C, Brown JB, Davis CA, Hillier L, Sisu C, Li JJ, et al.. 2014. Comparative analysis of the transcriptome across distant species. *Nature* **512**: 445–448.

Locke G, Tolkunov D, Moqtaderi Z, Struhl K, and Morozov AV. 2010. High-throughput sequencing reveals a simple model of nucleosome energetics. *Proc. Natl. Acad. Sci. U.S.A.* **107**: 20998–21003.

Mobius W, Osberg B, Tsankov AM, Rando OJ, and Gerland U. 2013. Toward a unified physical model of nucleosome patterns flanking transcription start sites. *Proc. Natl. Acad. Sci. U.S.A.* **110**: 5719–5724.

Moshkin YM, Chalkley GE, Kan TW, Reddy BA, Ozgur Z, van Ijcken WF, Dekkers DH, Demmers JA, Travers AA, and Verrijzer CP. 2012. Remodelers organize cellular chromatin by counteracting intrinsic histone-DNA sequence preferences in a class-specific manner. *Mol. Cell Biol.* **32**: 675–688.

Petesch SJ and Lis JT. 2008. Rapid, transcription-independent loss of nucleosomes over a large chromatin domain at Hsp70 loci. *Cell* **134**: 74–84.

Reichl LE. 2009. *A Modern Course in Statistical Physics, 3rd Revised and Updated Edition.* Wiley-VCH.

Rube HT and Song JS. 2014. Quantifying the role of steric constraints in nucleosome positioning. *Nucl. Acids Res.* **42**: 2147–2158.

Teif VB and Rippe K. 2009. Predicting nucleosome positions on the DNA: combining intrinsic sequence preferences and remodeler activities. *Nucl. Acids Res.* **37**: 5641–5655.

Teif VB and Rippe K. 2010. Statistical-mechanical lattice models for protein-DNA binding in chromatin. *J. Phys. Condens. Matter* **22**: 414105.

Teif VB and Rippe K. 2012. Calculating transcription factor binding maps for chromatin. *Brief. Bioinform.* **13**: 187–201.

Teves SS and Henikoff S. 2014. Transcription-generated torsional stress destabilizes nucleosomes. *Nat. Struct. Mol. Biol.* **21**: 88–94.

Vaillant C, Palmeira L, Chevereau G, Audit B, d'Aubenton Carafa Y, Thermes C, and Arneodo A. 2010. A novel strategy of transcription regulation by intragenic nucleosome ordering. *Genome Res.* **20**: 59–67.
